# Phenylacetonitrile (C_6_H_5_CH_2_CN) Ionic Liquid Blends as Alternative Electrolytes for Safe and High-Performance Supercapacitors

**DOI:** 10.3390/molecules25112697

**Published:** 2020-06-10

**Authors:** Flavien Ivol, Marina Porcher, Arunabh Ghosh, Johan Jacquemin, Fouad Ghamouss

**Affiliations:** Laboratoire de Physico-Chimie des Matériaux et des Électrolytes pour l’Énergie (PCM2E-EA 6299), Université de Tours, Parc de Grandmont, 37200 Tours, France; flavien.ivol@univ-tours.fr (F.I.); marina.porcher@univ-tours.fr (M.P.); jj@univ-tours.fr (J.J.)

**Keywords:** ionic liquids, phenylacetonitrile, alternative electrolyte, safety, supercapacitors

## Abstract

The increasing need in the development of storage devices is calling for the formulation of alternative electrolytes, electrochemically stable and safe over a wide range of conditions. To achieve this goal, electrolyte chemistry must be explored to propose alternative solvents and salts to the current acetonitrile (ACN) and tetraethylammonium tetrafluoroborate (Et_4_NBF_4_) benchmarks, respectively. Herein, phenylacetonitrile (Ph-ACN) has been proposed as a novel alternative solvent to ACN in supercapacitors. To establish the main advantages and drawbacks of such a substitution, Ph-ACN + Et_4_NBF_4_ blends were formulated and characterized prior to being compared with the benchmark electrolyte and another alternative electrolyte based on adiponitrile (ADN). While promising results were obtained, the low Et_4_NBF_4_ solubility in Ph-ACN seems to be the main limiting factor. To solve such an issue, an ionic liquid (IL), namely 1-ethyl-3-methylimidazolium bis [(trifluoromethyl)sulfonyl] imide (EmimTFSI), was proposed to replace Et_4_NBF_4_. Unsurprisingly, the Ph-ACN + EmimTFSI blend was found to be fully miscible over the whole range of composition giving thus the flexibility to optimize the electrolyte formulation over a large range of IL concentrations up to 4.0 M. The electrolyte containing 2.7 M of EmimTFSI in Ph-ACN was identified as the optimized blend thanks to its interesting transport properties. Furthermore, this blend possesses also the prerequisites of a safe electrolyte, with an operating liquid range from at least −60 °C to +130 °C, and operating window of 3.0 V and more importantly, a flash point of 125 °C. Finally, excellent electrochemical performances were observed by using this electrolyte in a symmetric supercapacitor configuration, showing another advantage of mixing an ionic liquid with Ph-ACN. We also supported key structural descriptors by density functional theory (DFT) and COnductor-like Screening Model for Real Solvents (COSMO-RS) calculations, which can be associated to physical and electrochemical properties of the resultant electrolytes.

## 1. Introduction

Supercapacitors have placed themselves in a unique position among all different emerging advanced energy storage systems because of their unique capability of delivering very high power, along with reasonably decent energy storage capabilities [1,2,3,4]. However, in order to achieve more applications for these devices, many efforts have been conducted during recent time to improve their energy density [5,6,7]. The energy density of a supercapacitor is expressed by the equation E = (1/2) CV^2^. Here, C is the capacitance, and V is the operational voltage of the supercapacitor. From this equation, it is very straightforward that increasing the operating voltage of the supercapacitor is an effective way to achieve higher energy density. Extension of operating voltage can be made by selecting an electrolyte, which can provide a higher electrochemically stable potential window [8].

Furthermore, the electrolyte is the sole medium, able to transport charges between the electrodes, i.e., the electrolyte is a key controlling factor for obtained current values within a supercapacitor. Therefore, one can genuinely appreciate that developing a suitable electrolyte is a key to control both the energy density and power density of a supercapacitor.

Developing a novel electrolyte is not a straightforward process. Beyond having a large potential window and high ionic conductivity, there are many other requirements that an electrolyte needs to meet to be classified as promising. An electrolyte must be chemically inactive and hence stable when in contact with the electrode materials and other device components, such as metallic current collectors, and the external packaging. The viscosity of the electrolyte is another crucial parameter. Viscosity controls the power density through two critical phenomena, ion diffusivity and pore accessibility. Besides, an electrolyte must have a large operating temperature range in the liquid state. This range is limited by the electrolyte melting point (or glass transition) and by its ebullition temperature (or decomposition). In other words, the prior knowledge of the thermal stability of electrolytes is vital and must be used to provide the operating temperature range of the energy storage devices. Furthermore, the volatility and flammability of the electrolyte play another key role in deciding the safety grades of a supercapacitor device. Controlling all of these parameters at the same time, makes development of an electrolyte very fascinating but a quite tricky job, which could be solely done by exploring a novel electrolyte chemistry.

There are wide varieties of alternative electrolytes available, such as solid-state electrolytes, or electrolytes in quasi-solid forms [9,10]. However, the present market is still dominated by liquid electrolytes. Liquid electrolytes are mainly classified based on the solvents and/or nature of salts used. To date, they are mainly categorized in three different types: aqueous, organic, and ILs [9,10]. While aqueous electrolytes are generally limited to a lower potential window up to 1.2 V (i.e., the free water decomposition potential), electrolytes based on ILs or those containing organic solvents can reach higher potential windows [9,10].

Among these three types of liquid electrolytes, ILs offer the highest potential windows, but at the same they are the most viscous in nature [11,12,13]. On the other hand, aqueous electrolytes can offer faster ion transport, however, that comes at the cost of compromised energy density, whereas, organic solvents can offer much higher potential windows compared to their aqueous counterparts. In addition, in organic solvents, ion diffusion is much faster compared to ILs, thanks to their comparatively lower viscosities. Therefore, organic solvents can provide an optimum performance when both energy and power delivery capabilities are concerned. Therefore, for obvious reasons, organic solvents have been the choice of solvent for commercial applications. For organic electrolytes, the most popular solvents are acetonitrile (ACN) and polycarbonates (PC) [14,15,16]. ACN-based electrolytes have lower viscosity and higher conductivity when compared to those of PC-based electrolytes and provide higher power performance of the supercapacitor devices [17,18]. The operating voltage of both the ACN- and PC-based EDLCs are limited to 2.5 V–2.7 V [8,9,10]. There have been many research efforts focused on further increasing the operation voltage of organic electrolytes; solvents like sulfones [19,20] or nitriles [21,22,23,24,25,26] were reported to have a higher operation voltage. Among nitrile solvents, ADN was explored as a possible alternative solvent for supercapacitors, improving flash point and operational window [22,23,25]. However, ADN also has a relatively high melting point (i.e., 2 °C) [27], which limits its use in low temperature operations. Thus, this short survey highlights that existing electrolytes either can deliver high power performances with compromised safety (e.g., ACN–based electrolytes), or higher energy density and fire safety, but with compromised mobility and thermal stability at low temperature (e.g., ADN-based electrolytes). Therefore, it is very difficult to identify an electrolyte which can fulfill well round application needs, i.e., which can work at sub-ambient temperatures, presenting a good mobility, low flammability, and wide working potential window. Hence, this attracted numerous numbers of research efforts focused in developing novel electrolytes with requested properties. The aim of this work is centralized on the utilization of Ph-ACN as an alternative solvent for the formulation of supercapacitor-based electrolytes. Thus, two series of alternative electrolytes based on this solvent were selected. The first series was obtained by dissolving the Et_4_NBF_4_ benchmark salt in Ph-can, whereas, the second series of electrolytes was obtained by mixing the Ph-ACN with an IL, namely 1-ethyl-3-methylimidazolium bis[(trifluoromethyl)sulfonyl] imide (EmimTFSI) based on our previous work on dinitrile [23].

These novel blends exhibited a relatively high potential window stability up to 3 V; while, it is noteworthy that this 3 V potential window was obtained in a symmetric supercapacitor configuration, i.e., without any mass balancing between positive and negative electrodes to extend the operating voltage of the supercapacitor [22]. Besides, each optimized electrolyte has shown significantly improved fire safety compared to ACN-based electrolytes, which is reflected by significantly higher flash points compared to those ACN based electrolytes. This is in fact associated to the low flash point of ACN (2 °C), making its electrolytes highly flammable, even at room temperature. Whereas, such an issue will be theoretically impossible by substituting ACN by Ph-ACN during the electrolyte formulation as its flash point (102 °C) is much higher. In addition, each selected Ph-ACN electrolyte is able to be used even under low temperature supercapacitor operations, unlike other proposed alternatives of ACN, such as ADN. By comparing these two series of Ph-ACN-based electrolytes, that containing the IL performed even better than that formulated using Et_4_NBF_4_, in terms of both charge storage capacity and rate capability, while maintaining all other excellent properties intact. Finally, all experimental data were supported by density functional theory (DFT) and COnductor-like Screening Model for Real Solvents (COSMO-RS) calculations to collect key structural descriptors, which may be associated to physical and electrochemical properties of the resultant electrolytes.

## 2. Results and Discussion

### 2.1. Formulation of Et_4_NBF_4_ Salt-Based Electrolytes in Nitrile Solvents

**Figure 1:**
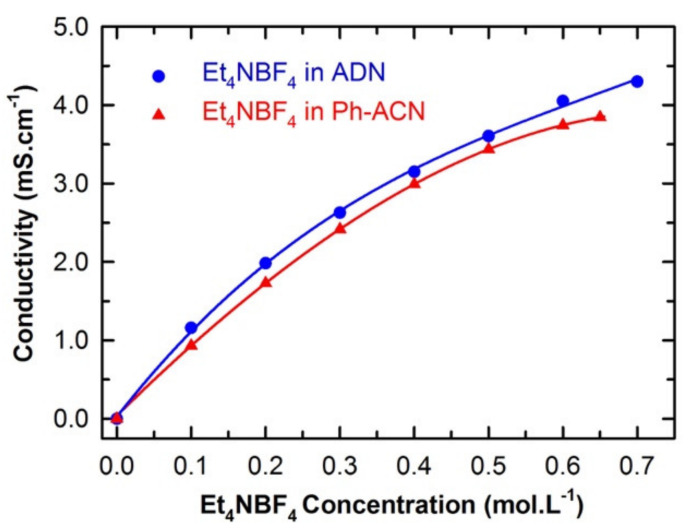
Comparison between the conductivities of Ph-ACN and ADN based electrolytes as a function of the Et_4_NBF_4_ salt concentration up to its solubility limit at 25 °C.

Figure 1 presents the dependency of the conductivity of the proposed electrolyte (Et_4_NBF_4_ salt in Ph-ACN) as a function of the salt concentration (at 25 °C), up to its saturation limit, which was found to be up to a concentration of 0.65 M (see Table S2 of ESI). This salt solubility limit is lower than that reported value in ACN (i.e., 1.68 M at 20 °C) [28], but this limit is very close to that determined for another alternative solvent, e.g., adiponitrile, ADN (0.7 M at 25 °C), in similar conditions [22]. The very limited solubility of Et_4_NBF_4_ could limit device performance by lowering ion conduction and increasing the serial resistance, especially at high power operation when significant concentration polarization could occur.

This relatively low solubility of Et_4_NBF_4_ in each solvent could be, in part, explained in terms of the salt ion pair dissociation energy, ΔEd. While the knowledge of other key parameters like the implications of crystal polymorph and solvent solvating power could provide a broader picture [29,30]. Herein, ΔE_d_ was calculated by the following methodology reported by Scheers et al. [31], stated in Equation (1):

ΔE_d_ = (E_cation_ + E_anion_) − E_ion pair_ (1)

Furthermore, as shown in the Table S1 of the Electronic Supporting Information (ESI), for the ions, Et_4_N^+^ (COSMO volume = 203.2902 Å^3^) and BF_4_^−^ (COSMO volume = 72.8506 Å^3^), the charge is highly localized on their specific surface; therefore, strong Coulombic and hydrogen bonding interactions between these ions are expected, leading to a higher ion pair dissociation energy of the Et_4_NBF_4_ (ΔE_d_ = 307.2 kJ·mol^−1^), than those observed in the case of classical ILs like Pyr_13_TFSI (ΔE_d_ = 299.0 kJ·mol^−1^ [32]), or Pyr_13_FSI (ΔE_d_ = 295.3 kJ·mol^−1^ [32]) or the EmimTFSI (ΔE_d_ = 297.3 kJ·mol^−1^ as reported in Table S1 of ESI of this article). The relatively higher ion pair dissociation energy of the Et_4_NBF_4_ salt can also explain its higher melting point and also to its relatively lower solubility in either ACN, ADN or Ph-ACN [23]. Furthermore, the Et_4_NBF_4_ salt solubility order in the selected solvents (i.e., ACN > AND~Ph-ACN), can also be associated to the volume of each solvent and its ability to solvate each ion in the solution as these solvents present mostly similar partial charge distribution on their cyano groups (see Table S1 of ESI). Solvates’ structural parameters can be estimated by determining the COSMO volume and sigma surface of selected solvents and ions, which can be also used to probe then their miscibility. By following this methodology, and using the data reported in Table S1 of ESI, one can expect that the solubility of Et_4_NBF_4_ in Ph-ACN (COSMO Volume = 159.2185 Å^3^) similar or slightly lower than in ADN (COSMO Volume = 155.6710 Å^3^). The highest salt solubility in ACN can be associated to its lower COSMO Volume (64.0469 Å^3^), and also to its lower distribution of charges in the van der Waals-type interactions region (e.g., the region from −0.010 e.Å^−2^ to +0.010 e.Å^−2^ in each sigma profile). The situation is however different for the two other nitriles. Furthermore, as previously demonstrate by our group [33], the maximum number of solvent molecules surrounding the Et_4_N^+^ cation could be determined by combining COSMO-RS and DFT calculations as a function of the solvent structure.

As reported in Table 1, these calculations demonstrate that six molecules of ACN can coordinate the Et_4_N^+^ cation, while a coordination number higher than three or four molecules could not be achieved for Ph-ACN or ADN, as another extra molecule will be solely localized in the second solvation shell. Therefore, the Et_4_N^+^ coordination number increases as follows: ACN (six molecules) >> ADN (four molecules) > Ph-ACN (three molecules). This result could be also used as a probe of the rate of solubility of Et_4_NBF_4_ in these solvents.

In the light of such an analysis along with conductivity data reported in Figure 1, one can see that both Ph-ACN- and ADN-based electrolytes exhibit a continuous increase in conductivity with increasing the salt concentration until the solubility limit. In other words, at the salt saturation, the maximum conductivity is reached at value close to 4 mS.cm^−1^ at 25 °C for 0.65 M of Et_4_NBF_4_ in Ph-ACN (see Table S3 of ESI). Furthermore, it appears that the conductivity of the saturated ADN-based electrolyte is slightly higher than that containing the Ph-ACN. This may be explained by comparing their salt solubility limit shown in Figure 1 and their Et_4_N^+^ coordination number as reported in Table 1.

**Figure 2:**
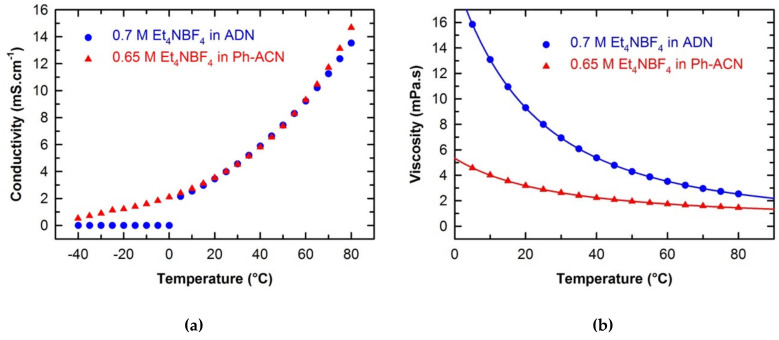
Temperature dependences on the conductivity (**a**) and the viscosity (**b**) of investigated electrolytes.

The conductivities of the ADN- and Ph-ACN-based electrolytes, were determined in a temperature range of −40 °C to 80 °C as shown in Figure 2a. Both electrolytes exhibited nearly identical conductivity values for temperature higher than 20 °C (see Table S3 of ESI). However, by looking at the conductivity at lower temperatures (especially those below 0 °C,) one can clearly see that ADN-based electrolyte is not suitable anymore. Whereas, the Ph-ACN-based electrolyte still maintained a reasonably good ionic conductivity, demonstrating its potential as an alternative electrolyte for low temperature applications, even at −40 °C (with a residual conductivity of 0.52 mS·cm^−1^). This observation can be directly related to the melting point of the pure Ph-ACN, (Tm = −24 °C), which is much lower than that of ADN (Tm = +1.8 °C) as shown in Supplementary Figure S1 of the ESI. However, these solvents have a melting temperature higher than that of ACN (Tm = −45 °C). These results are in excellent agreement with those available in the literature [27]. Similar trend was then observed by comparing the DSC traces of the selected electrolytes, which both have a melting temperature higher than that of the benchmark electrolyte, as also shown in Figure S1 of the ESI. The residual conductivity observed in the case of the Ph-ACN-based electrolyte could be certainly associated to the coexistence of a solid and liquid sub-phases at low temperature like those observed in the case of eutectic mixtures. This is also consistent with the fact that no transition nor crystallization peak was observed on its DSC trace during the cooling step down to −60 °C, which is obviously not the case of the ADN sample as expected from conductivity data reported in Figure 2a.

To depict in detail the differences in their transport properties, the viscosity of selected electrolytes and solvents were also determined as a function of temperature from 5 °C to 80 °C as shown in Figure 2b. All data are tabulated in Table S3 of the ESI. By looking at these data, it is obvious that regardless of the solvent structure and temperature, the addition of salt induces a viscosity increase. As shown in Figure 2b, the viscosity of each electrolytic solution decreases with the temperature, as expected. Furthermore, regardless of the temperature, Ph-ACN-based electrolyte is less viscous than the ADN blend. This observation seems logical as both solvents present a nearly identical Et_4_N^+^ solubility (Figure 1) and the pure ADN is more viscous than Ph-ACN over the whole temperature range studied (Table S3 of ESI). More interestingly, the viscosity difference between these two electrolytes seems to be even more pronounced for temperature below 30 °C, demonstrating an interesting feature of the Ph-ACN solvent. However, both alternative electrolytes are more viscous than the current benchmark, which could be also related to the very low viscosity of ACN (Table S3 of ESI).

By combining the data represented in Figure 2a,b, one can clearly expect, for temperatures higher than 0 °C, that the Walden product (W = Λ·×·η) of the 0.65 M Et_4_NBF_4_ in Ph-ACN electrolyte will be lower than that of the 0.7 M Et_4_NBF_4_ in ADN. For example, at 25 °C, W_Ph-ACN_ = 0.18 S·P·cm^2^·mol^−1^ and W_ADN_ = 0.46 S·P·cm^2·^mol^−1^ for Ph-ACN and ADN-based electrolytes, respectively. From these Walden product values; one can thus argue that the ET_4_NBF_4_ is more dissociated in ADN than in Ph-ACN. This could be then related to the difference observed between their Et_4_N^+^ coordination numbers as reported in Table 1. However, on the one hand, the lower viscosity of Ph-ACN vs. ADN, promotes faster ion diffusion and hence 0.65 M Et_4_NBF_4_ in Ph-ACN exhibits similar conductivity (Figure 2a) despite of a lower ion dissociation than in ADN-based electrolyte. Moreover, no noticeable residual ionic conductivity of ADN-based electrolyte was observed for temperature below its melting temperature (see Figure S1 of ESI), which is in very good agreement with conclusions already reached in the literature [24]. On the other hand, good residual ionic conductivities for Ph-ACN were obtained, even at −40 °C. Thus, by assessing combined thermal and transport properties, one can clearly expect that this Ph-ACN-based electrolyte can be used in energy storage devices even at very low temperatures down to −30 °C, which is obviously not the case of ADN.

### 2.2. Comparative Charge Storage Performance of Ph-ACN and ADN Based Electrolytes

**Figure 3:**
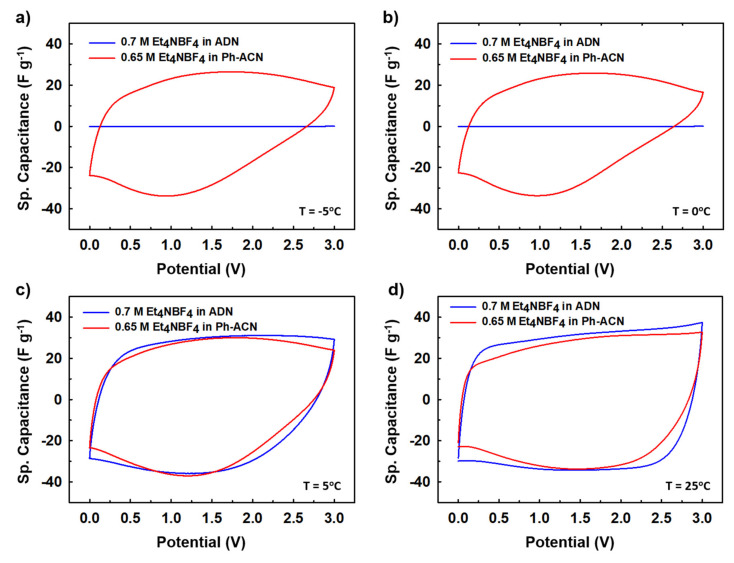
Comparative charge storage performances (CV performances) at different temperatures, (**a**) −5 °C, (**b**) 0 °C, (**c**) 5 °C, and (**d**) 25 °C, for the selected Ph-ACN and ADN based electrolytes.

**Figure 4:**
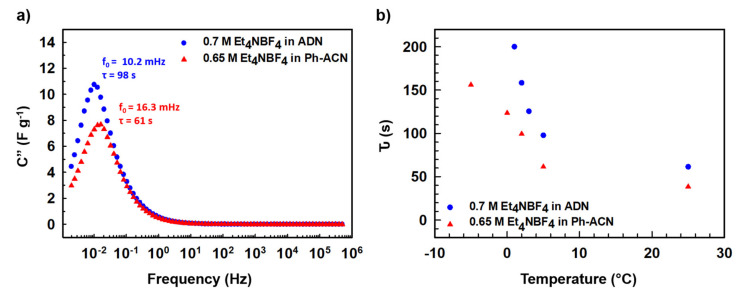
(**a**) Evolution of the imaginary part of the capacitance (C”) of selected Ph-ACN and ADN based electrolytes at 5 °C with frequency. (**b**) Temperature dependence on the characteristic time constants for both electrolytes.

**Figure 5:**
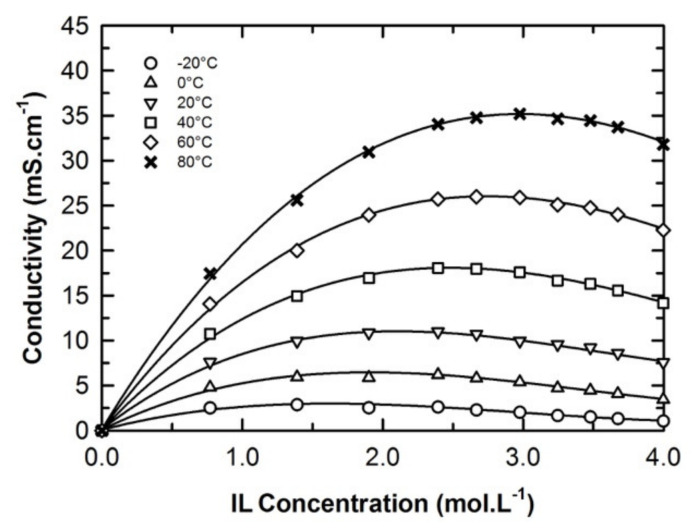
Concentration and temperature dependences in the conductivity of the EmimTFSI and Ph-ACN binary mixture.

In the light of the above observations, we then decided to further assess selected alternative electrolytes in terms of their electrochemical performances using Teflon Swagelok^®^ cells in an AC/AC symmetric two-electrode configuration. Therefore, CVs were run at 25 °C, 5 °C, 0 °C and then −5 °C. As shown in Figure 3, and unsurprisingly, cycling is not possible at temperature below 0 °C (Figure 3a,b) when the device is filled with the ADN-based electrolyte, comforting the conclusions above-mentioned thanks to DSC and conductivity data. It is interesting to also note that, at 5 °C (Figure 3c) and 25 °C (Figure 3d), both electrolytes performed very similarly as comparable capacitance values close to (25.1 or 27.2) F·g^−1^ at 5 °C and (26.2 or 29.9) F·g^−1^ at 25 °C are observed using the Ph-ACN or ADN-based electrolytes, respectively. More importantly, even though with reduced temperature, the shape of the CV curves had deviated from a perfect rectangular shape to a more oval shape, Ph-ACN blend is able to provide significant amount of charge storage even at 0 °C (Figure 3b) or −5 °C (Figure 3a) with a capacitance value close to 20 F·g^−1^ even at −5 °C. This observation is a direct result of reasonably good ionic mobility of the Ph-ACN-based electrolyte, even at low temperature, as shown in Figure 2a. Along with this, we can see better charge storage performance from the more rectangular CV shapes, with increasing temperatures. This phenomenon can be explained by increased ionic conductivity and decreased viscosity of the electrolyte, which improved ionic mobility and the wettability of the electrodes. In order to investigate further on the ionic mobility of the both Ph-ACN and ADN-based electrolytes in an AC/AC symmetric configuration, EIS measurements were run at 25 °C but also at low temperatures (e.g., 5 °C, 3 °C, 2 °C and 1 °C) to provide a wider picture. Characteristic time constants were then calculated from the following equation: τ (s) = 1/f_0_; where f_0_ is the characteristic frequency, expressed in Hz (e.g., the peak frequency in the Bode plot shown in Figure 4a). At 5 °C, as depicted by looking at Figure 4a, a faster ions transport is reached in the Ph-ACN blend, with a time constant close to 61s, than in ADN-based electrolyte (time constant ~98 s) as previously assumed from collected viscosity data. Figure 4b represents a plot for different characteristic time constants for both the Ph-ACN and ADN-based electrolytes at different temperatures. Unsurprisingly, the characteristic time constant of both electrolytes increases by decreasing the temperature, which was expected by looking at the temperature dependences on their transport properties (Figure 2). However, with the respect of conditions applied, it is worth noticing that in the case of the ADN-based electrolyte, the characteristic time constants seem to be more affected by the temperature changes than those collected for Ph-ACN blend. This indicates for ADN-based electrolyte, with decreasing temperatures, increased difficulties in ion diffusions, were more severe compared to that of Ph-ACN.

### 2.3. Formulation of Ph-ACN Based IL Mixture

After testing the Ph-ACN with the benchmark Et_4_NBF_4_ salt, promising results, especially when compared to ADN were obtained. However, two main drawbacks were also noticed, namely: (i) a limited Et_4_NBF_4_ solubility in Ph-ACN solvent and (ii) a higher melting temperature of the optimized blend compared to the ACN-benchmark electrolyte. To solve such issues, we then decided to substitute the Et_4_NBF_4_ salt by an ionic liquid, EmimTFSI. As per our expectations, from our previous study on ADN + EmimTFSI [23], this mixture was also found to be fully miscible over the whole composition range. This opportunity giving thus the possibility to formulate and characterized highly concentrated IL solutions, up to 4.0 M, corresponding to the pure IL (see Tables S4 and S5 of ESI). This excellent solubility contrasts the relatively limited Et_4_NBF_4_ solubility in Ph-ACN. Such a difference could be explained by the structure of these salts as the EmimTFSI is based on asymmetrical ions leading to weaker cation-anion interactions and thus to a lower dissociation energy than Et_4_NBF_4_ (see Table S1 of ESI). This difference could be also associated by the much lower melting point of the EmimTFSI, Tm = −18 °C (see Figure S1 of ESI), compared to that expected for the Et_4_NBF_4_ salt (380 °C, [34]). Furthermore, as reported in Table S6 of the ESI, DFT calculations demonstrated that 4 molecules of Ph-ACN can coordinate the Emim^+^ cation, while as above-mentioned only 3 molecules are observed in the case of the Et_4_N^+^-Ph-ACN cluster (see Table 1). This difference could be explained by the presence of π–π interactions between the Emim^+^ and Ph-ACN, which is obviously not the case between the Et_4_N^+^ and Ph-ACN. Additionally, the lower volume (155.7999 Å^3^ vs. 203.2902 Å^3^) and the wider delocalization of the charge (see sigma profiles reported in Table S1 of ESI) on the Emim^+^ compared to Et_4_N^+^ may be also other associated factors explaining the complete miscibility between the IL and Ph-ACN

The optimization of the electrolyte formulation was then driven by the collection of conductivity data as a function of the composition at various isotherms as shown in Figure 5 (see also Table S4 of the ESI). During this work, nine blends with a concentration close to 0.8, 1.4, 1.9, 2.3, 2.7, 3.0, 3.2, 3.5, or 3.7 M of EmimTFSI in Ph-ACN were thus prepared at 25 °C. Their conductivity values were measured and then systematically compared to those for the pure IL (i.e., 4.0 M) from −20 °C to 80 °C as shown in Figure 5. As expected, regardless of the temperature, for each isotherm, the conductivity increases until reaching a maximum value prior to decreasing down to the conductivity of the pure IL (4.0 M). However, it is very interesting to observe that at very low temperatures, i.e., those lower than 0 °C, the influence of the electrolytes formulation on the conductivity is less significant than that observed at higher. Moreover, the location of the highest conductivity values seems to be temperature dependent moving from approx. 2.0 M (low temperature) to 3.0 M (high temperature). By taking into consideration different formulation factors including the shift of these conductivity maxima with the temperature, conductivity values and electrolyte cost (driven by the quantity of IL added in the solution), we then decided to further analyze the 2.7 M EmimTFSI in Ph-ACN as a potential alternative electrolyte for supercapacitors.

### 2.4. Electrochemical Charaterization of Ph-ACN/EmimTFSI Blend

The electrochemical stability windows (ESW) of investigated Ph-ACN-based electrolytes and pure IL were then determined with a three-electrode cell using platinum disc as the working electrode. The result is depicted in Figure S2 of ESI and in Table S7 of the ESI. The ESW for the 0.65 M Et_4_NBF_4_ in Ph-ACN (ESW = 4.29 V) seems to lower than that reported either for the 1 M Et_4_NBF_4_ in ACN (ESW = 5.0 V) [35] or 0.7 M Et_4_NBF_4_ in ADN (ESW = 5.55 V) [24]. To support this experimental observation, DFT calculations were then done to determine the HOMO and LUMO energies for each species involved as shown in Table S8 of ESI. By comparing the HOMO and LUMO energies of ADN or ACN with those determined for the Ph-ACN, it appears that the presence of the phenyl moiety decreases the polarization charge range of the solvent considerably, as the Ph-ACN has the highest HOMO (−7.178 eV) and the lowest LUMO (−0.871 eV) energies of the investigated solvents. Nevertheless, by comparing the HOMO and LUMO energies of each solvent with those determined for the Et_4_NBF_4_ salt, one can clearly see that highest HOMO level is observed for the salt (localized on its anion, as expected from energies determined for each individual ion), while its LUMO energy (localized on its cation, as also expected from results of individual ions) is slightly higher (−0.746 eV) than that of the Ph-ACN (−0.871 eV). In other words, based on collected HOMO-LUMO energies, one can thus expect to reach a slightly lower electrochemical windows by mixing the Et_4_NBF_4_ salt with Ph-ACN than with the two other solvents. Furthermore, as also shown in Figure S2 of ESI, the ESW for the pure EmimTFSI (ESW = 4.40 V) seems to be lower than that of the 2.7 M EmimTFSI in Ph-ACN (ESW = 4.94 V). This could be again associated to HOMO-LUMO energies differences (see Table S8 of ESI, HOMO = −7.377 eV and LUMO = −1.951 eV), but also by the presence of π-π interactions between the [Emim]^+^ and Ph-ACN, which could increase the electrochemical stability of both the cation and Ph-ACN.

**Figure 6:**
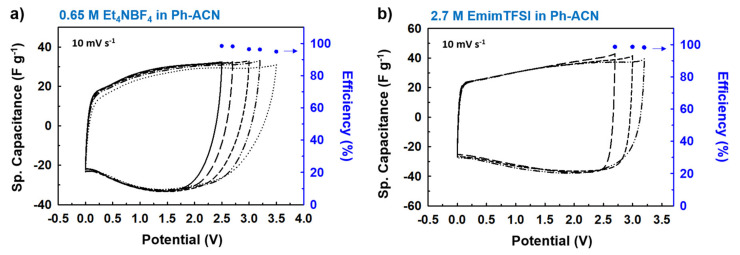
Charge discharge performances and corresponding efficiencies of the device vs. applied potential window, through CV curves at a scan rate of 10 mV·s^−1^ for selected Ph-ACN blends; (**a**) 0.65 M Et_4_NBF_4_ in Ph-ACN and (**b**) 2.7 M EmimTFSI in Ph-ACN. As shown in the Figure 6a,b, regardless of the applied potentials, very similar CV curves with very similar specific capacitances were obtained in each case. Then, the charge-discharge efficiencies were systematically investigated for all the applied potentials, and excellent efficiencies—ca. ~94–98% for 0.65 M Et_4_NBF_4_ in Ph-ACN and ~98% for 2.7 M EmimTFSI in Ph-ACN—were determined from the respective CV curves. This behavior clearly indicates that 0.65 M Et_4_NBF_4_ in Ph-ACN electrolyte is stable over a very high potential window up to 3.2 V, whereas the 2.7 M EmimTFSI in Ph-ACN can solely work within a potential window of 3.0 V.

However, in real applications, an electrochemically stable potential window should be reported from a 2-electrode cell configuration and using activated carbon as the specific surface and porosity of the electrode also influences the ESW. Figure 6a,b represent all CV curves (scan rate of 10 mV·s^−1^) for different applied working potentials on the Swagelok cells-based supercapacitor device in symmetrical configuration, by using the following electrolytes: 0.65 M Et_4_NBF_4_ in Ph-ACN and 2.7 M EmimTFSI in Ph-ACN.

**Figure 7:**
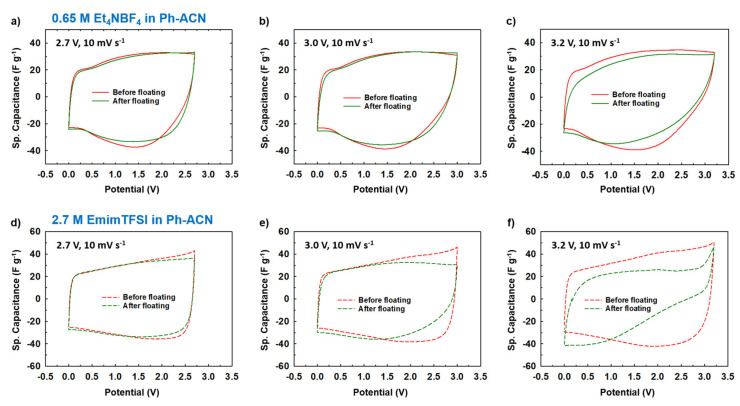
Estimation of the operative voltage of selected electrolytes from collected CVs before and after the floating test done at 2.7 V, 3.0 V or 3.2 V, for 0.65 M Et_4_NBF_4_ in Ph-ACN (**a**–**c**) and 2.7 M EmimTFSI in Ph-ACN (**d**–**f**).

**Figure 8:**
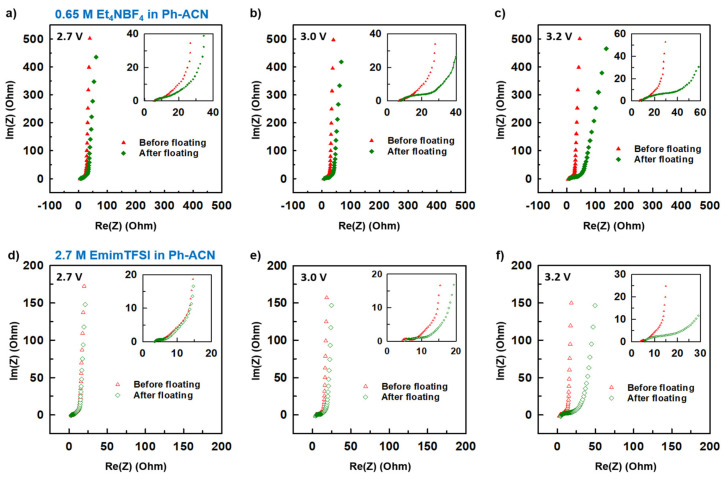
Estimation of the operative voltage of selected electrolytes from collected Nyquist plots before and after the floating test done at 2.7 V, 3.0 V or 3.2 V, for 0.65 M Et_4_NBF_4_ in Ph-ACN (**a**–**c**) and 2.7 M EmimTFSI in Ph-ACN (**d**–**f**).

However, solely based on data reported in Figure 6, it is very difficult to conclude on a realistic value of the operative voltages of an electrolyte. This could be assessed by cycling the electrolyte over a longer period of time and under a fix potential (i.e., floating tests). Therefore, in order to conclude on their stability, floating tests using Swagelok cells for different potential windows of 2.7, 3.0 and 3.2 V were realized in each case. Thus, the operating potential windows of selected electrolytes was assessed by comparing their CVs (Figure 7). Electrochemical impedance spectroscopy (EIS, Figure 8) was also recorded before and after floating tests.

More precisely, Figure 7a–c and Figure 7d–f present collected CV curves (10 mV·s^−1^) before and after floating tests for 0.65 M Et_4_NBF_4_ in Ph-ACN and for 2.7 M EmimTFSI in Ph-ACN, respectively. As shown in Figure 7a,b, in the case of the Et_4_NBF_4_-based electrolyte, almost no difference between the before- and after-floating CV curves is observed for both 2.7 V and 3.0 V potential windows. Similar stability was also observed for both 2.7 V and 3.0 V potential windows in the case of the 2.7 M EmimTFSI in Ph-ACN (Figure 7d,e). However, regardless of the electrolyte, we observed a little dip in the values of current while applied voltage is fixed at 3.2 V (Figure 7c,f). In each case, the after-floating CV curve for 3.2 V potential window is indeed significantly poorer compared to before-floating CV. Similarly, Figure 8a–c and Figure 8d–f present the impact of floating on Nyquist plots of selected Ph-ACN blends for different operating potentials, 2.7, 3.0 and 3.2 V. As expected, and shown in the Figure 8, negligible changes on impedance spectra are observed when the potential window is lower than 3.0 V. Whereas, for higher potential windows, especially at 3.2 V, noticeable deviations between before and after floating tests are observed. The main changes can be noticed at the medium to low frequency regions. This indicates that the storage mechanism is altered, possibly by side reactions due to the electrolyte decompositions at higher voltages. It is worth noticing, that the electrolyte resistances, which are determined from the intersections of the impedance spectra with the real axis of the Nyquist plot at the highest frequency, increase after floating, and were also found to be increased with increasing the applied voltage. This indicates gradual increase in the electrolyte resistances with decompositions of the electrolytes (Table S9 of the ESI). Nevertheless, from the Table S9, one can see that, all the cells maintained their capacitive behaviors even after the floating tests, evidenced by the almost vertical lines in the Nyquist plots at the low frequency’s domain. However, there was no loss in the maximum specific capacitances, which can be determined from the imaginary part of the impedance at the lowest frequency (Figure S5 of the ESI). The specific capacitances were in fact observed to be little higher for both the electrolytes for the after the floating cases for 2.7 and 3.0 V. This little enhancement in the values can be attributed to the possible increase in the wettability of the cells during the floating tests, and therein the long-time holding at the high voltages. Whereas, a slight decrease was observed with both the electrolytes at the 3.2 V (Table S9 of the ESI). However, the capacitance drops more quickly with the increase of the frequencies for all samples after the floating test. Moreover, the amplitude of this drop increases with the applied voltage, which is in accordance with the increase of the serial resistance discussed above.

This before and after floating tests comparison clearly indicates that, to prevent their decomposition, both electrolytes could not be used at this voltage or higher. In other words, these results confirmed that in the case symmetric supercapacitor configuration, studied Ph-ACN electrolytes have an operative voltage of 3 V, which is larger than both the PC- (2.7 V) and ACN- (2.5 V) benchmark electrolytes [8,9,10].

### 2.5. Comparative Charge Storage and Safety Performances of the Formulated Electrolytes

**Figure 9:**
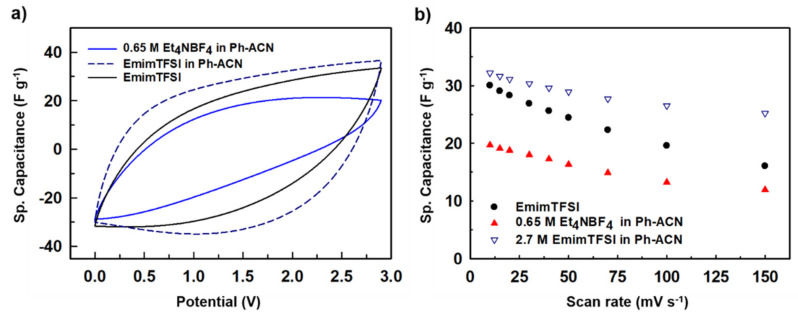
(**a**) Comparative CV plots and (**b**) comparative rate capabilities (Sp. capacitance vs scan rates) for proposed two electrolytes and the IL alone.

Figure 9a shows CV curves determined in a symmetrical AC two-electrode cell at a potential window of 2.9 V i.e., 100 mV lower than the upper value above-mentioned with a scan rate of 100 mVs^−1^ for the 0.65 M Et_4_NBF_4_ in Ph-ACN, 2.7 M EmimTFSI in Ph-ACN and the pure EmimTFSI for comparison purpose. From this figure, it appears that the electrolyte has the best CV shape, which is more rectangular than the shape observed with the two other electrolytes. Furthermore, as displayed in Figure 9b, this electrolyte provides also the best electrochemical performances. This observation emphasizes, in fact, the importance of the formulation as the 2.7 M EmimTFSI in Ph-ACN electrolyte is a result of a perfect optimization between a high ions concentration (compared to 0.65 M Et_4_NBF_4_ in Ph-ACN) and a relatively low viscosity (compared to the pure EmimTFSI–see Tables S4 and S5 of the ESI).

Walden products were then determined from collected physical properties (using data tabulated in Tables S3–S5 of the ESI) as close to W = 0.18, 0.48 and 0.75 S·P·cm^2^·mol^−1^ at 25 °C for 0.65 M Et_4_NBF_4_ in Ph-ACN, 2.7 M EmimTFSI in Ph-ACN, and EmimTFSI, respectively. As mentioned above, the highest Walden product does not necessarily mean the highest storage performance, as exemplified herein by comparing the results obtained using the pure EmimTFSI and its blend with Ph-ACN. In this case, the high viscosity of EmimTFSI (33.85 mPa·s at 25 °C) plays a key role in limiting the capacitance value. In fact, the use of high viscosity electrolyte not only limits its ions diffusion considerably during charge-discharge process, but also their ability to completely enter within the porous network, hence a very poor wettability of the electrode limits the total charge storage capability. Whereas, the 0.65 M Et_4_NBF_4_ in Ph-ACN electrolyte has lowest viscosity (2.88 mPa·s at 25 °C), however due the low concentration of the salt, and thus of ions in the electrolyte, lower capacitance was observed in caparison with ionic liquid-based electrolytes. Nonetheless, when the IL is dissolved in the Ph-ACN higher concentration of ions and result in an increase charge storage capability as shown in Figure 9a. Furthermore, the addition of Ph-ACN in the EmimTFSI induces a decrease of the viscosity from 33.85 mPa·s to 10.4 mPa·s at 25 °C, leading to an easier transportation of the ions through the porous electrodes and better wettability. This also mean a lower serial resistance in the device as it is evidenced by the more rectangular shape of the CV curves. Figure 9b shows the rate performance of the supercapacitors using these different electrolytes, and as expected from the above discussion, one can observe that 2.7 M EmimTFSI in Ph-ACN not only exhibited highest charge storage capabilities, but also the best rate performances, thanks to its well-balanced physical properties (such as σ = 12.7 mS·cm^−1^ at 25 °C) compared to the other two tested electrolytes. All investigated electrolytes were tested for fire safety by determining their flash point. Prior presenting, collected data tabulated in Table 2, the flash point of the pure solvents was determined to be close to 2 °C, 102 °C and 160 °C for the ACN, Ph-ACN and ADN, respectively. This order seems to follows that expected from their normal boiling point as highlighted in Figure S4 of ESI. By comparing the value of the Ph-ACN (102 °C) with those determined for Ph-ACN blends electrolytes higher flash points close to 110 °C and 125 °C were obtained by for both the electrolytes containing the Et_4_NBF_4_- and EmimTFSI, respectively. As shown in Table 2, both flash point values are higher than that determined for benchmark electrolyte, highlighting that the Ph-ACN-based electrolytes are safer, in the case of a short-circuit, than ACN-based electrolyte (Fp = 5 °C). However, ADN-based electrolyte; like the selected 0.7 M Et_4_NBF_4_ in ADN, seems to be even more safe than Ph-ACN but one can recall that this electrolyte cannot be used for low temperatures operations (i.e., T < 0 °C).

Furthermore, to provide a broader picture of the operating temperature range (i.e., delimited by the liquid temperature range), TGA measurements of investigated IL, solvents and electrolytes were then determined as shown in Figure S3 of ESI. From this analysis, no clear difference was observed when comparing the TGA curves between each pure solvent and its corresponding electrolyte containing the Et_4_NBF_4_ salt. This observation could be directly linked to the very low salt concentration in solution. However, as reported in Table 2, the upper temperature limit of utilization of this series of electrolytes is ranked as follows ACN (45 °C) < Ph-ACN (130 °C) < ADN (184 °C) showing another interesting feature of using Ph-ACN solvent during the electrolyte formulation. More interestingly, a wider operational temperature window was even observed within the 2.7 M EmimTFSI in Ph-ACN electrolyte as shown in Figure S3 of the ESI. We have obtained an operation temperature range of −60 °C to 131 °C for the IL and Ph-ACN blend, and this low temperature performance of a supercapacitor device is very important for cold weather applications, such as for automobiles, for wearable electronics, and for energy grids stored at colder places, at the polar regions of earth, and also for device operations in outer space.

### 2.6. Quantification of the Relative Performance of Electrolytes through a Relative Performance Index Code

As shown during this work, the selection of an alternative electrolyte is not a simple task, which must be re-thought as an electrolyte depends upon many independent parameters, which are again very different in nature. This selection, in fact, further impacts on the overall performances of the whole device. Therefore, a well-thought performance tool was established to eventually help quantifying and then selecting an alternative electrolyte able to substitute current benchmarks. For this reason, a kind of Quantitative Structure-Property Relationship (QSPR) so called, herein, a relative performance index, was developed with respect of the current benchmark electrolyte based on Et_4_NBF_4_ in ACN.

On the one hand, as the electrochemical performance of a given electrolyte is mainly controlled by two most important parameters, namely, the operating voltage windows (OVW) and the conductivity (σ) of the electrolyte. The first one directly influences the specific energy stored by a supercapacitor and its value is directly proportional to the square of operation potential of the supercapacitor. Whereas the power performance of a supercapacitor can be assumed to be proportional to the conductivity of the electrolyte since the major serial resistance in a supercapacitor comes from the electrolyte resistance. For this reason, herein, the two following QSPR factors, indicating energy storage ability and power performance of an electrolyte, were firstly identified as critical:

(i) Specific energy factor (SEF), which was determined by the relation [SEF] = [OVW]^2^
(ii) Specific power factor (SPF), which was set as the conductivity of the electrolyte determined at 25 °C; thus [SPF] = [σ]

On the other hand, two other criteria, related to the working/operational temperature range of a given electrolyte and more importantly its safety, were recognized as crucial for the selection of an alternative solvent. As either the upper limit of the working operational range or the flash point (Fp) of a given electrolyte could be selected, for safety reason the latter were preferred. Furthermore, as nothing is more important than the safety, and to mimic the SEF, the high temperature operation factor (HTOF) value was thought as the square of flash point. However, no confusion is possible for the low temperature range as solely the melting temperature (Tm) of the given electrolyte could be used. For this reason, herein, the two additional QSPR factors, indicating the lowest temperature ability and safety of an electrolyte, were then identified as crucial:

(iii) High temperature operation factor (HTOF), which was determined by the relation [HTOF] = [Fp]^2^
(vi) Low temperature operation factor (LTOF), which was determined by the relation [LTOF] = [Tm]

Note that for mathematical reasons, all temperatures were converted to Kelvin. Moreover, SEF has been determined from the operating voltage window obtained using symmetrical supercapacitor without any mass balancing of the electrodes. It is also believed that this operating voltage can be further increased with mass balancing of the positive and negative electrode.

Then each relative QSPR parameter of a given electrolyte, reported in Equation (2), was simply determined by calculating the ratio between each of its four selected factors by those expected for a reference electrolyte presenting an OSW of 2.7 V, a conductivity of 48.70 mS·cm^−1^ comparable to the benchmark ACN electrolyte, a flash point of 60 °C and a melting point of −30 °C. In other words, this ideal electrolyte was used herein as a standard reference electrolyte (SRE) during the setup of the proposed QSPR. For example, the relative specific energy factor (r-SEF) of an electrolyte denoted ‘A’ is determined as r-SEF = [(OSW)_A_/(OSW)_SRE_] ^2^. The other three relative factors were also determined using a similar manner, leading to, for a given electrolyte called “A”:

(a) Relative specific energy factor (r-SEF); which, relatively quantifies the stored energy ability of the targeted electrolyte compared to the SRE: r-SEF = [(OSW)_A_/(OSW)_SRE_]^2^.
(b) Relative specific power factor (r-SPF); which, relatively quantifies the power delivery capability of the targeted electrolyte compared to the SRE: r-SPF = [(σ)_A_/(σ)_SRE_].
(c) Relative high temperature operation factor (r-HTOF); which, relatively quantifies the maximum temperature to be set with the respect of the device safety between the electrolyte and the SRE: r-HTOF = [(Fp)_A_/(Fp)_SRE_]^2^.
(d) Relative low temperature operation factor (r-LTOF); which, relatively quantifies the minimum temperature to be set with the respect of the melting temperature of the electrolyte and the SRE. As this temperature must be the lowest as possible this relative factor was set as r-HTOF = [(Fp)_SRE_/(Fp)_A_]. Indeed, by using this expression, a factor with a value higher than 1 will indicate a better performance than the SRE as the three other relative factors.

Therefore, the so-called relative performance index (r-PI) with respect to the standard reference electrolyte (SRE) is thus determined as follows:

r-PI = [(r-SEF) x (r-SPF) x (r-HTOF) x (r-LTOF)] (2)

However, it is worth mentioning that Equation (2) could be improved by simply adding other important properties (such as the vapour pressure, long-term stability and cyclability, etc.) or by weighting each factor differently, as any QSPR method. However, by using the proposed methodology, the above-mentioned parameters/factors were systematically calculated for all investigated electrolytes as reported in Table 3.

From Table 3, it is worth noticing that, the best relative performance index (r-PI) was achieved for the ACN-based electrolyte (r-PI = 0.756) mainly because of its very high conductivity, followed by 2.7 M EmimTFSI in Ph-ACN with a r-PI of 0.457. However, it is important to highlight herein that this methodology is proposed to helping decision on the selection of alternative electrolytes by considering not solely its performances but also its safety impact with the respect of a so-called ideal electrolyte. Indeed, Ph-ACN can provide safe operations when it is used to formulate alternative electrolytes over a wide range of temperature, thanks to its higher boiling temperature (Bp = 234 °C), inducing a lower vapor pressure inducing less environmental hazards (e.g., inhalation) than ACN (Bp = 82 °C). By depicting all data collected during this work along with those presented in Table 2 and Table 3, and all other associated results, one can really appreciate that Ph-ACN-based electrolytes present some interesting characteristics and could be designated as safer electrolytes which can potentially substitute the ACN-benchmarks used to date.

## 3. Materials and Methods

### 3.1. Materials

Phenylacetonitrile (Ph-ACN, CAS number: 140–29–4) was an industrial by-product of unknown purity, kindly provided by Axyntis Group (Paris, France). Prior to being used, the as-received Ph-ACN was distilled under reduced pressure. Acetonitrile (ACN, ≥99.0%, CAS number: 75–05–8), adiponitrile (ADN, ≥99.0%, CAS number: 111–69–3) and tetraethylammonium tetrafluoroborate salt (Et_4_NBF_4_, ≥99.0%, CAS number: 429–06–1) were purchased from Sigma-Aldrich (St. Louis, SM., USA). 1-Ethyl-3-methylimidazolium bis(trifluoromethylsulfonyl)imide (EmimTFSI, 99.9%, CAS number: 174899–82–2) was purchased from Solvionic (Toulouse, France). All commercial solvents were also distilled under reduced pressure prior to be stored in a glove-box, while the salts were used as received as both have a low water level (below 10 ppm) as determined by Karl-Fisher titration (899 Titrando, Metrohm, Herisau, Switzerland). To avoid any contaminations from the atmosphere, all the products were stored in a glove box (model number: MB-200B, MBraun, Munich, Germany) under an argon atmosphere (O2 < 0.1 ppm and H2O < 0.1 ppm). Activated carbon-based electrodes coated on aluminum current collectors were purchased from Samwha Capacitor (Seoul, South Korea). These electrodes were composed of 85 wt.% of activated carbon as active material, and 15 wt.% of additives with a total mass loading of 7 mg·cm^−2^. Whatman GF/C glass microfiber filters (0.26 mm thickness, 1.2 µm pore size) were used as separator in the supercapacitor.

### 3.2. Preparation of the Electrolytes and Electrochemical Cells

Organic solvent-based electrolytes were prepared on a mass basis by dissolving a known quantity of Et_4_NBF_4_ in ADN, ACN and Ph-ACN, in order to obtain concentrations ranging from 0 to 0.7 M at 25 °C. Please note that the molar concentration was then calculated thanks to the prior knowledge of the density of each electrolytic solution (density values are given in Tables S3 and S5 of the ESI). Similarly, EmimTFSI and Ph-ACN blends were also prepared by mass, with a Ph-ACN mass fraction ranged from 0 to 1 (with an increment of 0.1), corresponding to an IL molar concentration increasing from 0 M to 4 M. To prevent any contamination with residual water from the atmosphere, all organic and ionic liquid-based electrolytes were prepared and stored in a glove box under an Ar atmosphere. For similar reasons, supercapacitor devices were also assembled in the glove box using Teflon Swagelok^®^ cells in an AC/AC symmetric two-electrode configuration. A separator soaked with 120 µL of electrolyte solution was placed between the two electrodes. Prior assembling, electrodes (0.785 cm^2^) and separators were dried under reduced pressure at 80 °C during 4 h in order to ensure removal of residual moisture and also to allow a non-contamination of the glovebox environment. For the three-electrode cell configuration, platinum was used as working electrode (WE, 2.01 mm^2^) and a glassy carbon electrode as a counter electrode (CE, 7.07 mm^2^). A silver wire, whose potential was calibrated vs. ferrocenium/ferrocene (Fc^+^/Fc) redox couple was used as a quasi-reference electrode.

### 3.3. Measurement Methods

Conductivity measurements were carried out from −40 °C to 80 °C using a Multichannel Conductivity Meter, based on frequency response analyser (MCS 10, Bio-Logic, Clermont-Ferrand, France). Measurements were carried out using sealed cells with Pt parallel-plate-electrodes, to protect the samples from air exposure. Prior to any measurements, conductivity cells were calibrated using aqueous KCl solution (standard conductivity solutions; 147 μS·cm^−1^, 1413 μS·cm^−1^, and 12.88 mS·cm^−1^) at 25 °C.

Density and viscosity measurements were performed as a function of the temperature using a digital vibrating tube densimeter (mode 60/602, Anton Parr and a rolling-ball viscometer (Lovis 2000 M/ME, Anton Parr, Craz, Republik Österreich), respectively. Data acquisitions were realized at thermal equilibrium with successive increments of 5 °C within the range of 5 °C to 80 °C. Prior to any measurements, instruments were cleaned with water, ethanol and acetone and dried with dehumidified air. Density and viscosity values of distilled water were then measured to check the calibration of the apparatus.

Thermal phase transitions were recorded with a Differential Scanning Calorimeter (DSC 4000, Perkin Elmer, Waltham MA, USA) coupled with an Intracooler SP VLT 100. Aluminum pans containing the electrolyte were prepared and sealed in the glove box under an argon atmosphere. Electrolytes were first cooled down to −60 °C, kept for at least 5 min at −60 °C and then heated up to 30 °C at a scan rate of 5 °C·min^−1^, under a nitrogen atmosphere. For each electrolyte, two successive scans were carried out in order to improve the reproducibility of the measurement.

The flammable behaviour of the electrolytes was studied with an automated closed cup flash point tester (Setaflash Series 8, Stanhope-SETA, Hillsborough St, NC, USA) with a temperature range of 2 °C to 300 °C (scan of 2 °C·min^−1^)

Electrolyte thermal stability were carried out with a Sensor Thermogravimetric Analyser (STA 6000, Perkin Elmer) under a nitrogen atmosphere within the range of 25 °C to 700 °C at a scan rate of 5 °C·min^−1^ in alumina (Al_2_O_3_) pans. Decomposition temperatures reported from TGA data are the onset, as determined from the step tangent.

Electrochemical measurements including operating voltage experiments, floating tests and Electrochemical Stability Windows (ESW) studies, were carried out on a Versatile Multichannel Potentiostat (VMP3, Bio-Logic) piloted by an EC Lab interface. To determine operating voltages, cyclic voltammetry (CV) measurements were carried out applying a scan rate of 10 mV·s^−1^ and gradually increasing the potential limit. The cell operating voltage was determined for a potential whose coulombic efficiency of the CV is greater than 98.5%.

Floating tests were performed at 23 °C in a temperature-controlled room. These tests consist of following steps; (i) charging the device with a constant current density of 1 A·g^−1^ until the potential limit is reached, (ii) maintaining this potential limit value for 12 h, and (iii) discharging the device to 0 V with a current density of −1 A·g^−1^. Before and after floating tests, CV, galvanostatic charge-discharge (GCD) and electrochemical impedance spectroscopy (EIS) measurements were realized to check the stability of the supercapacitor devices. CV measurements were done at 10 mV·s^−1^ while GCD profiles were realized at 1 A·g^−1^. EIS measurements were realized at 0 V vs. ref in the range of 500 kHz to 2 mHz with 10 mV of amplitude.

ESW measurements were studied by CV with a scan rate of 1 mV·s^−1^ starting from the open circuit voltage (OCV) to positive potential until a strong current response was detected. The potential is then scanned in the same way from the OCV to reduction potential after cleaning the surface of the WE. The ESW was defined when the oxidation and reduction currents reached ±1 mA·cm^−2^.

### 3.4. Calculation Methods

Calculations of the sigma surface and structure descriptors, such as the COSMO volume, HOMO and LUMO energies, were completed using the Turbomole 7.0 programme package [36]. Before being able to visualize the 3D structure of each species using TmoleX (version 4.1.1), all structures were optimized in the gas phase with a convergence criterion of 10^−8^ Hartree, using HF-6 311G**++ basis set, followed by DFT calculations combining the resolution of identity (RI) approximation, [37,38] within the Turbomole 7.0 programme package using the B3LYP function with the def2-TZVPD basis set [39,40]. The resulting optimized structures were then used as inputs in the COSMOconfX programme (version 4.0) to generate the conformers of each species using the BP-TZVPD-COSMO+GAS basis set. The COSMO volume (Å^3^), the sigma profile and the sigma surface of each species were then determined and viewed using the COSMOthermX software, which is based on the COSMO-RS (COnductor-like Screening MOdel) computation technique. The combination of TmoleX and COSMOthermX software (version 19.0.5) was then used to understand the differences in structure, charge distribution and volume of the three solvents, EmimTFSI and Et_4_NBF_4_ investigated during this study and to understand the specific cation-solvent interactions in solution as a function of the number of solvent molecules in the first solvation shell by following the methodology previously described by our group [33,41,42].

## 4. Conclusions

In the light of its relatively low melting temperature, high boiling and flash points, Ph-ACN was investigated as a potential alternative solvent able to replace ACN when formulating EDLC-based electrolytes. During their formulation, the benchmark Et_4_NBF_4_ and EmimTFSI were selected as the conductive salts. Thanks to an in-depth formulation and characterization exercise, two sets of alternative organic electrolytes, namely 2.7 M EmimTFSI in Ph-ACN and 0.65 M Et_4_NBF_4_ in Ph-ACN were then selected. During this optimization, some differences between the selected salts were observed, firstly only a limited solubility of the Et_4_NBF_4_ in Ph-ACN could be achieved at 25 °C, while the IL–Ph-ACN mixture was found to be fully miscible. Secondly, a larger electrochemical stability windows was observed by selecting the 2.7 M EmimTFSI in Ph-ACN than the Et_4_NBF_4_ blend. In addition, even if the two electrolytes exhibit a much higher flash points than the 1 M Et_4_NBF_4_ in ACN (Fp = 5 °C), a safer electrochemical device could be achieved using the 2.7 M EmimTFSI in Ph-ACN (Fp = 125 °C) than the 0.65 M Et_4_NBF_4_ in Ph-ACN (Fp = 110 °C). Furthermore, this EmimTFSI blend has optimized physical properties (liquid range temperature, transport properties and ionicity) with the respect of pure IL salt outperforming those obtained for 0.65 M Et_4_NBF_4_ in Ph-ACN. More importantly, these two Ph-ACN-based electrolytes have a higher operating stability window of 3.0 V in each case, leading to higher amount of charge storage performance, than 1 M Et_4_NBF_4_ in ACN benchmark. This work also highlights the main difficulties on the selecting alternative electrolytes, as compromises must be done. For example, selected Ph-ACN-based electrolytes have a lower flash point than 0.7 M Et_4_NBF_4_ in ADN (Fp = 163 °C); while they both have a much lower melting point, at least −30 °C, than the ADN alternative (Tm = −4 °C). Therefore, in order to provide more conclusive remarks on the comparative performances of electrolytes with respect an ideal electrolyte presenting desired properties in term of conductivity, operating stability window, melting temperature and flash point, a QSPR so-called relative performance index was proposed. This has again thrown promising light on both the Ph-ACN-based electrolytes in terms of all-round electrochemical charge storage performance features, including fire safety and reduced environmental hazards.

## Figures and Tables

**Table 1:** Optimized structure of the investigated Et_4_N^+^ cation + ACN or ADN or Ph-ACN clusters.

ACN	ADN	Ph-ACN
6 ACN + 1 Et_4_N^+^ 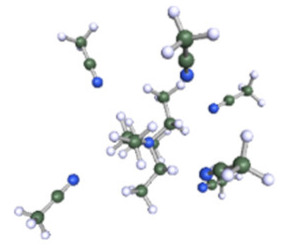	4 ADN + 1 Et_4_N^+^ 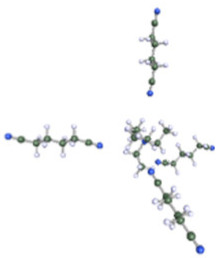	3 Ph-ACN + 1 Et_4_N^+^ 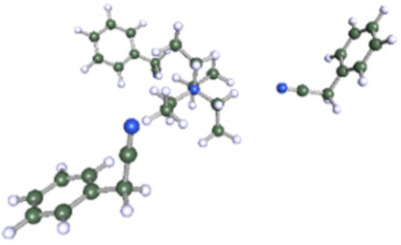
7 ACN + 1 Et_4_N^+^ 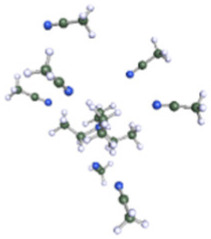	5 ADN + 1 Et_4_N^+^ 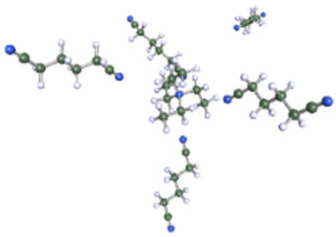	4 Ph-ACN + 1 Et_4_N^+^ 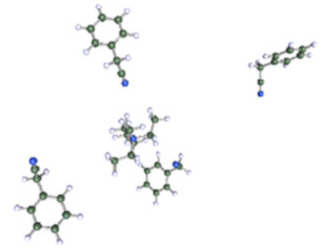

**Table 2:** Physical properties of different electrolytes.

Electrolyte	η at 25 °C (mPa·s)	σ at 25 °C (mS·cm^−1^)	Operating Temperature Range (°C)	Flash Point (°C)
1.0 M Et_4_NBF_4_ in ACN	0.639	48.7	−49 °C to 45 °C	5
1.0 M Et_4_NBF_4_ in ADN	7.996	3.98	−4 °C to 184 °C	163
0.65 M Et_4_NBF_4_ in Ph-ACN	2.884	4.00	−30 °C to 130 °C	110
2.7 M EmimTFSI in Ph-ACN	10.40	12.57	−60 °C to 131 °C	125

**Table 3:** Calculated parameters/factors for a relative performance index code.

Electrolyte	OSW (V)	σ (mS·cm^−1^)	Fp (K)	T_m_ (K)	r-SEF	r-SPF	r-HTOF	r-LTOF	r-PI
Standard reference electrolyte (SRE)	2.7	48.70	333.15	243.15	1	1	1	1	1
1.0 M Et_4_NBF_4_ in ACN	2.7	48.70	278.15	224.15	1.000	1.000	0.697	1.085	0.756
0.65 M Et_4_NBF_4_ in Ph-ACN	3.0	4.0	383.15	243.15	1.111	0.082	1.323	1.000	0.121
2.7 M C_2_mimTFSI in Ph-ACN	2.9	12.71	398.15	213.15	1.074	0.261	1.428	1.141	0.457

## Supplementary Materials

The following are available online, Figure S1: Heating traces of the DSC thermograms of the pure solvents and electrolytes based on (a) ADN, (b) ADN, (c) Ph-ACN and (d) EmimTFSI, Figure S2: Electrochemical windows of the 0.65 M Et_4_NBF_4_ in Ph-ACN, EmimTFSI and 2.7 M EmimTFSI in Ph-ACN electrolytes, Figure S3: Thermogravimetric analysis (TGA) curves of the pure solvents and electrolytes based on (a) ADN, (b) ADN, (c) Ph-ACN and (d) EmimTFSI, Figure S4: Overview of the liquid range temperature of pure solvents and the operating temperature range of selected electrolytes. Each ★ represent the normal boiling point of pure solvent involved in a given electrolyte, Figure S5: Frequency response of the specific capacitances, before and after the floating tests, done at 2.7 V, 3.0 V and 3.2 V, for the 0.65 M Et_4_NBF_4_ in Ph-ACN (a-c), and 2.7 M EmimTFSI in Ph-ACN (d-f), Table S1: Structure, abbreviation, COSMO volume and sigma profile of each studied conformer, Table S2: Experimental conductivity data of Ph-ACN and ADN based electrolytes as a function of the Et_4_NBF_4_ salt concentration up to its solubility limit at 25 °C as measured in a glovebox environment, Table S3: Experimental physical properties as a function of the temperature of pure solvents and selected Et_4_NBF_4_-based electrolytes at 101 kPa, Table S4: Experimental conductivity data (σ/mS·cm^−1^) of investigated Ph-ACN + EmimTFSI blends as a function of the composition, expressed in IL mole fraction, *x*_IL_ and IL concentration in mol·L^−1^, and the temperature at 101 kPa, Table S5: Experimental physical properties as a function of the temperature of pure IL and 2.7 M EmimTFSI in Ph-ACN electrolyte at 101 kPa, Table S6: Optimized structure of investigated Emim^+^ cation + Ph-ACN clusters, Table S7: Electrochemical stability windows of selected electrolytes, Table S8: Structure, HOMO and LUMO energies of each selected solvent, ion and salt, Table S9. Electrolyte resistance (Rs), electrolyte series resistance (ESR), and specific capacitances (C), before and after floating tests for both the 0.65 M Et_4_NBF_4_ in Ph-ACN (a), and 2.7 M EmimTFSI in Ph-ACN (b)
