# Supplementary material for: Phenylacetonitrile (C6H5CH2CN) Ionic Liquid Blends as Alternative Electrolytes for Safe and High-Performance Supercapacitors
Source: Molecules. 2020 Jun 10;25(11):2697. doi: 10.3390/molecules25112697 (PMC7321299; doi:10.3390/molecules25112697)
Supplement: Supplementary file 1 [file molecules-25-02697-s001.pdf]

Phenyl-acetonitrile ($C_6H_5CH_2CN$) ionic liquid blends as alternative electrolytes for safe and high-performance supercapacitors.

Flavien Ivol, Marina Porcher, Arunabh Ghosh*, Johan Jacquemin, Fouad Ghamouss*

Laboratoire de Physico-Chimie des Matériaux et des Électrolytes pour l'Énergie (PCM2E-EA 6299), Université de Tours, Parc de Grandmont, 37200, Tours, France

* Correspondence: arunabh.ghosh@univ-tours.fr (A.G.); ghamouss@univ-tours.fr (F.G.)

Received: 14 May 2020; Accepted: 8 June 2020; Published: 10 June 2020

Electronic Supporting Information

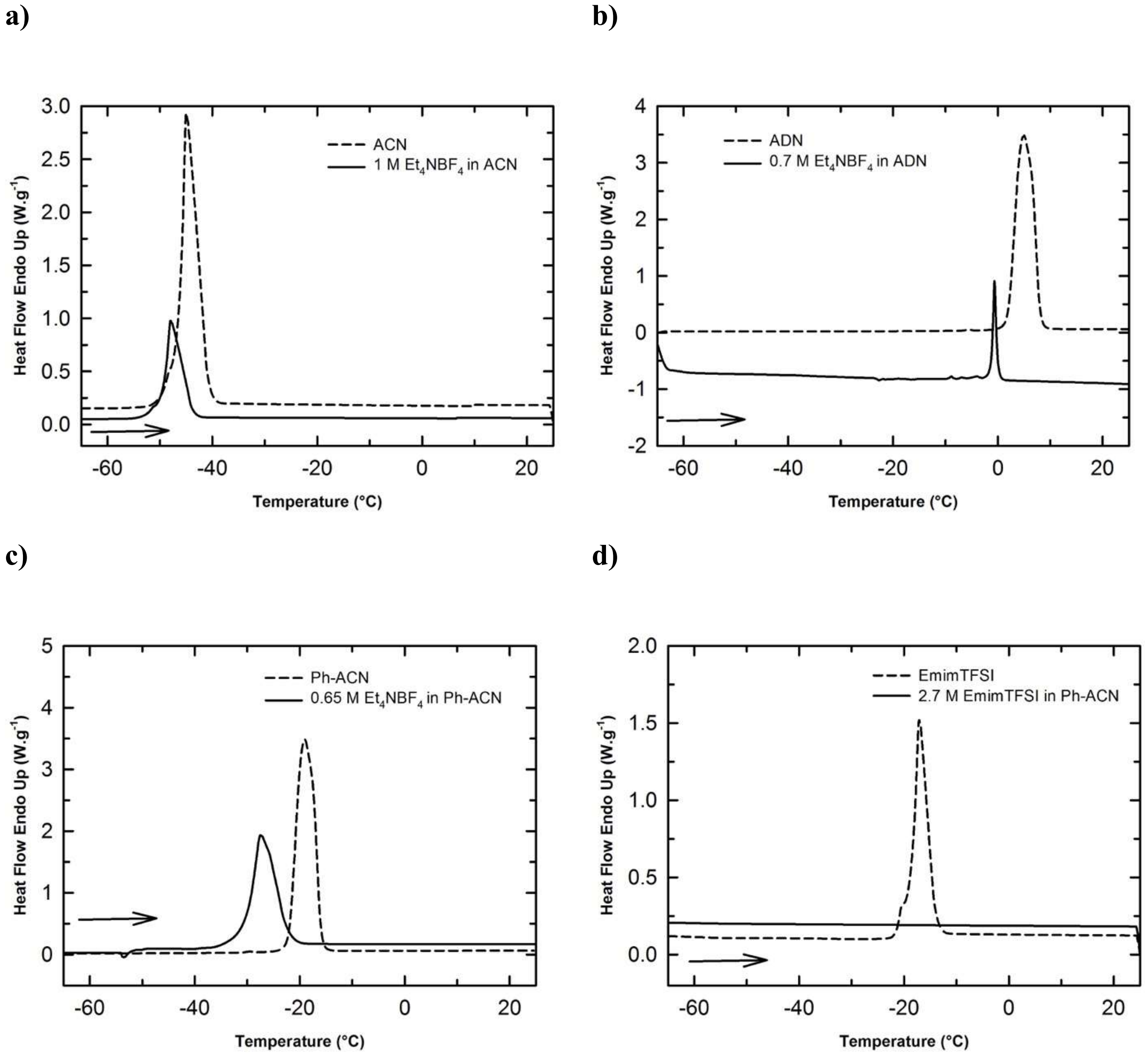

Figure S1. Heating traces of the DSC thermograms of the pure solvents and electrolytes based on (a) ADN, (b) ADN, (c) Ph-ACN and (d) EmimTFSI.

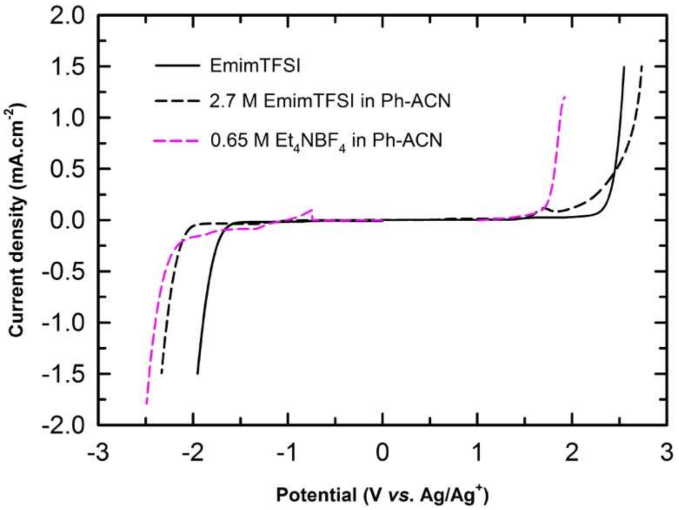

Figure S2. Electrochemical windows of the 0.65 M Et_4NBF_4 in Ph-ACN, EmimTFSI and 2.7 M EmimTFSI in Ph-ACN electrolytes.

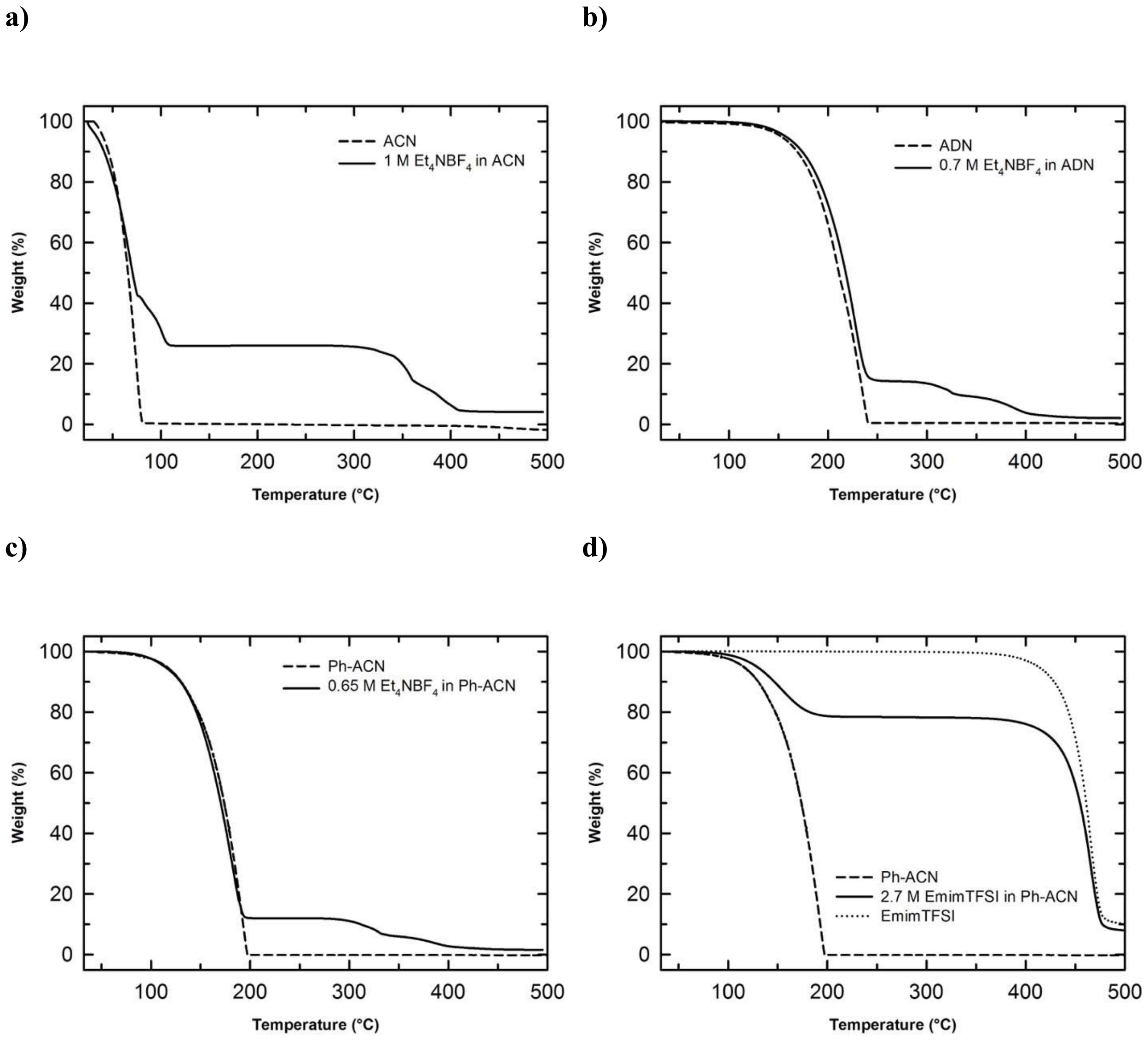

Figure S3. Thermogravimetric analysis (TGA) curves of the pure solvents and electrolytes based on (a) ADN, (b) ADN, (c) Ph-ACN and (d) EmimTFSI.

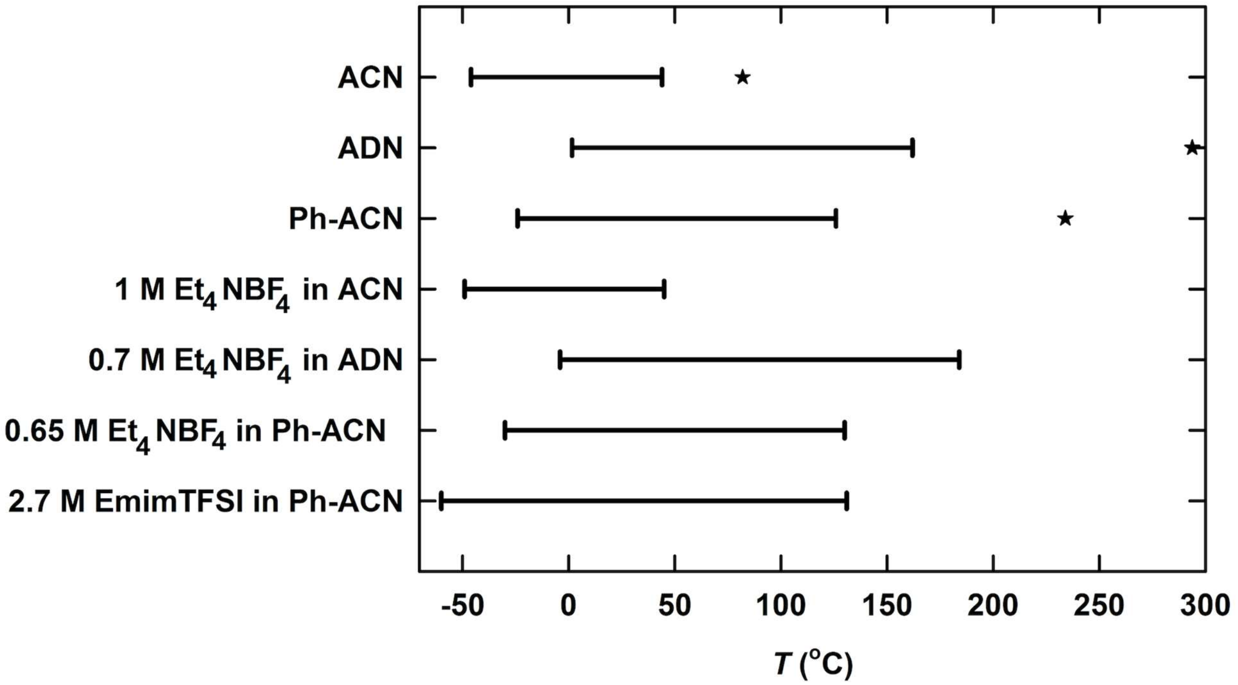

Figure S4. Overview of the liquid range temperature of pure solvents and the operating temperature range of selected electrolytes. Each ★ represent the normal boiling point of pure solvent involved in a given electrolyte.

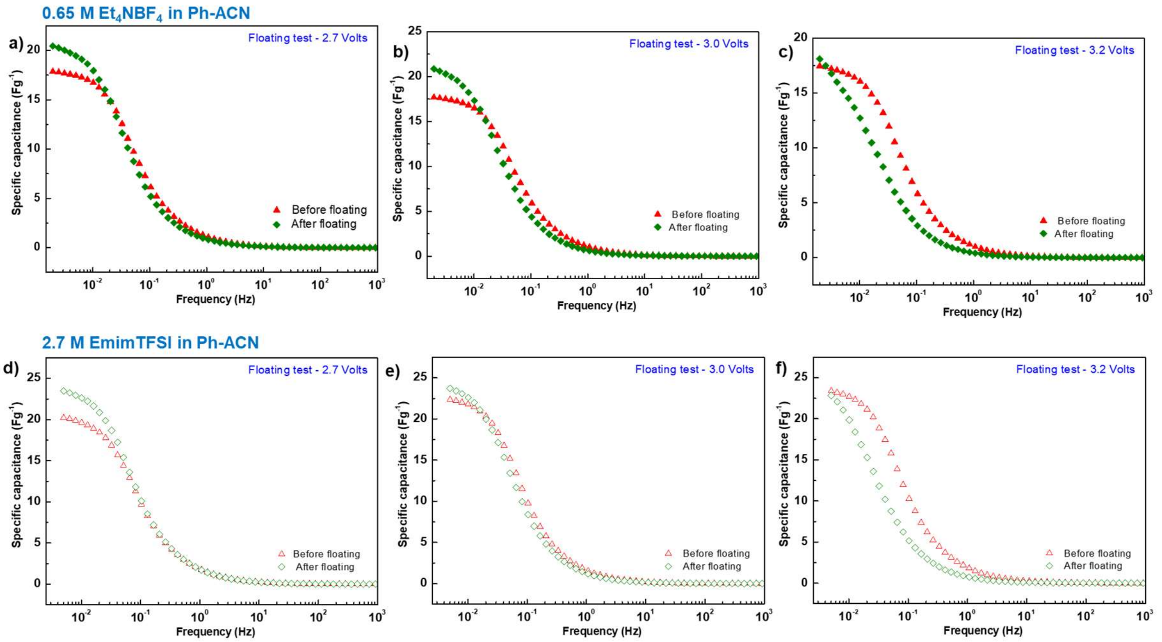

Figure S5. Frequency response of the specific capacitances, before and after the floating tests, done at 2.7 V, 3.0 V and 3.2 V, for the 0.65 M Et_4NBF_4 in Ph-ACN (a-c), and 2.7 M EmimTFSI in Ph-ACN (d-f)

Table S1. Structure, abbreviation, COSMO volume and sigma profile of each studied conformer.

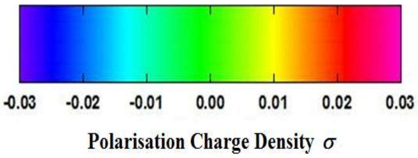	COSMO volume (Å^3)	Sigma profile
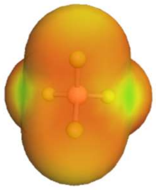<br>BF_4^-	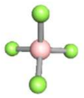<br>72.8506 Å^3	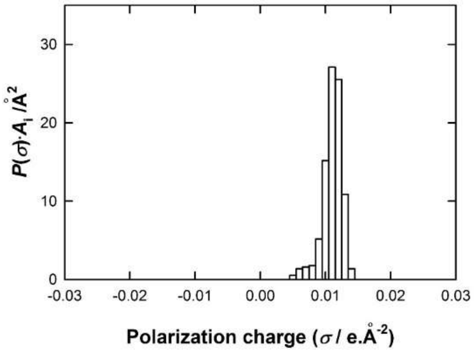
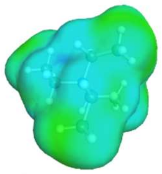<br>Et_4N^+	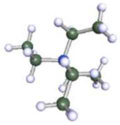<br>203.2902 Å^3	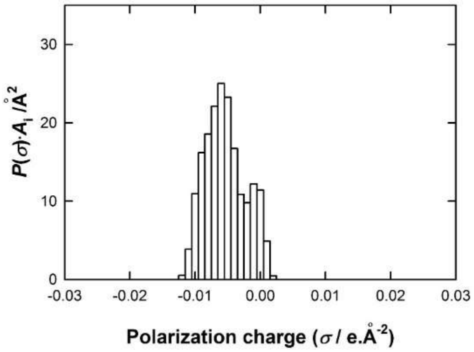
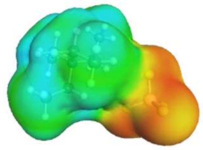<br>Et_4NBF_4<br>conformer 1<br>ΔE_d = 307.2 kJ·mol^{-1}	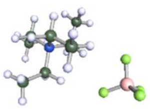<br>283.4462 Å^3	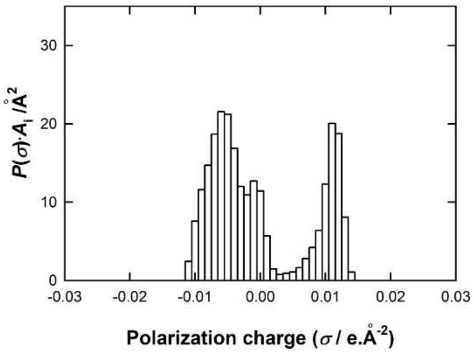

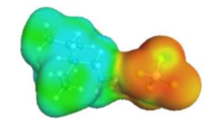<br>Et_4NBF_4<br>conformer 2	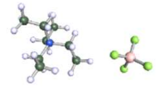<br>282.4163 Å^3	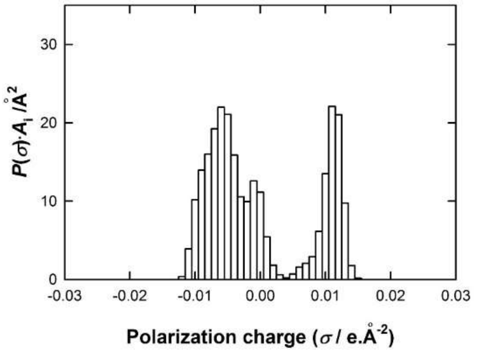
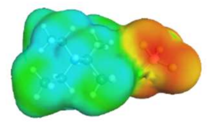<br>Et_4NBF_4<br>conformer 3	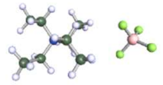<br>282.2207 Å^3	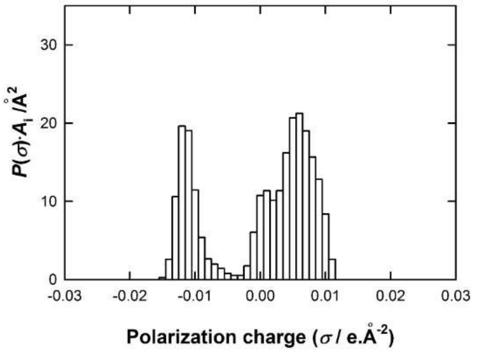
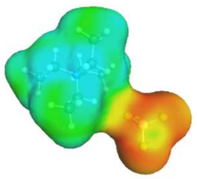<br>Et_4NBF_4<br>conformer 4	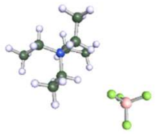<br>281.5999 Å^3	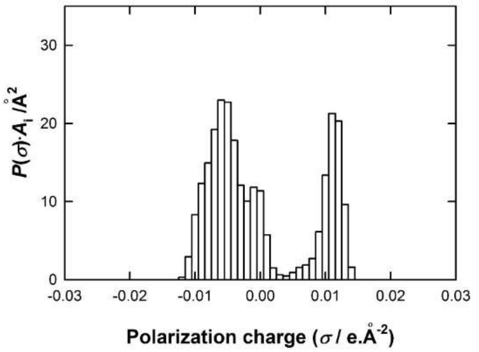
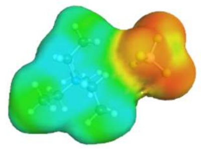<br>Et_4NBF_4<br>conformer 5	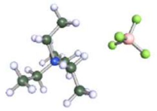<br>279.7787 Å^3	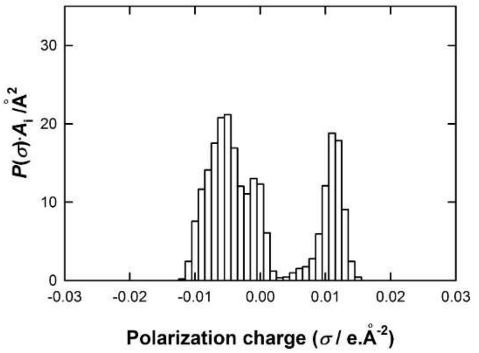

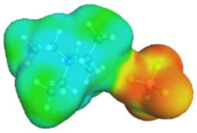<br>Et_4NBF_4<br>conformer 6	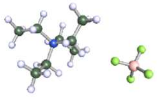<br>277.4122 Å^3	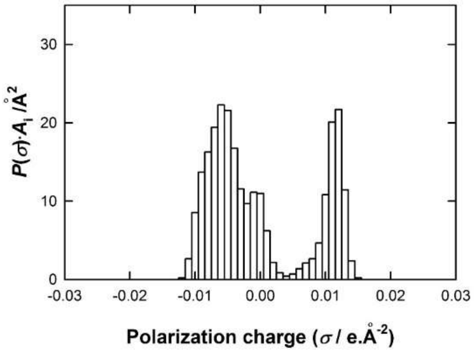
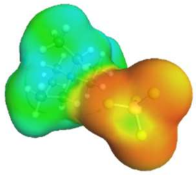<br>Et_4NBF_4<br>conformer 7	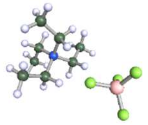<br>281.0190 Å^3	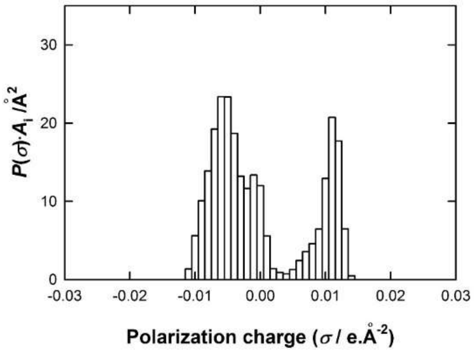
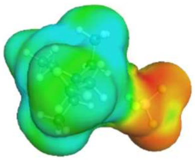<br>Et_4NBF_4<br>conformer 8	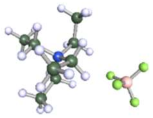<br>276.6653 Å^3	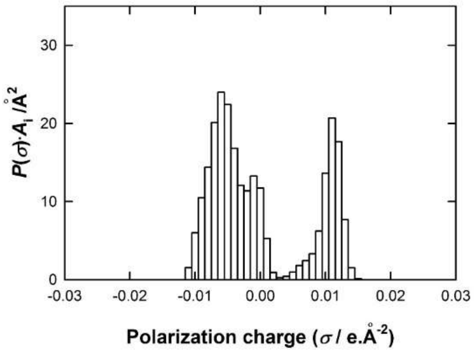
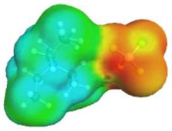<br>Et_4NBF_4<br>conformer 9	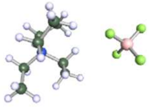<br>283.2684 Å^3	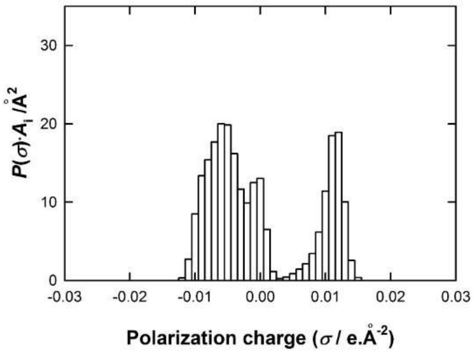

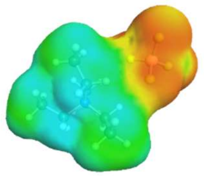 Et_4NBF_4 conformer 10	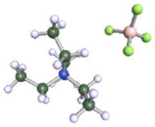 280.5492 Å^3	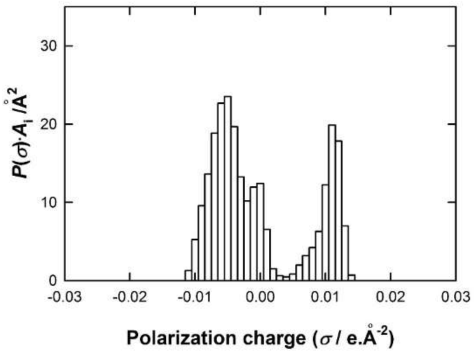
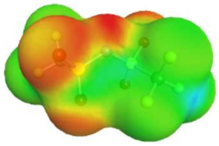 $TFSI^-$ conformer 1	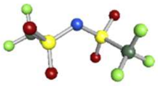 219.6735 Å^3	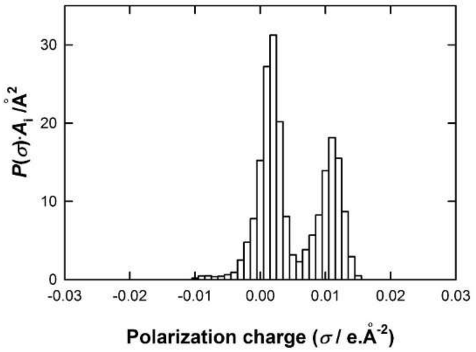
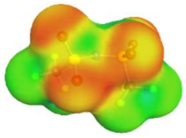 $TFSI^-$ conformer 2	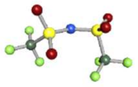 222.2341 Å^3	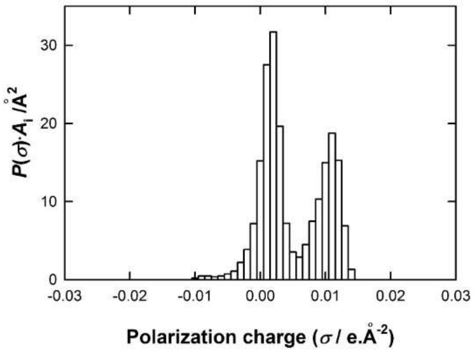
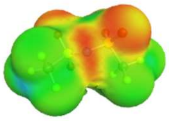 $TFSI^-$ conformer 3	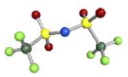 222.5364 Å^3	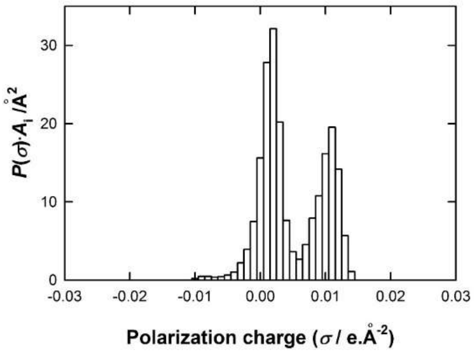

<table>
<tr>
<td>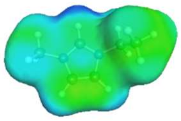
Emim$^+$
conformer 1</td>
<td>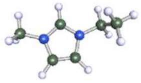
155.7999 Å^3</td>
<td>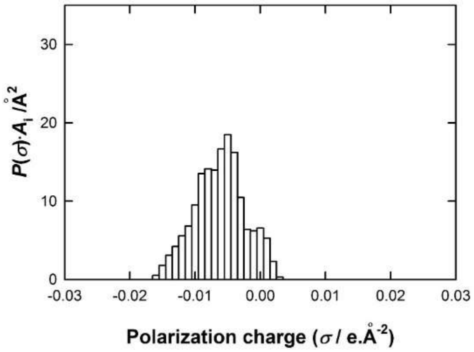
</td>
</tr>
<tr>
<td>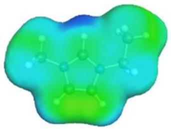
Emim$^+$
conformer 2</td>
<td>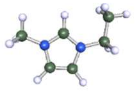
154.1699 Å^3</td>
<td>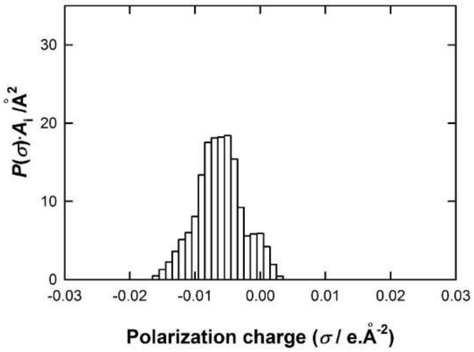
</td>
</tr>
<tr>
<td>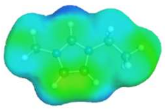
Emim$^+$
conformer 3</td>
<td>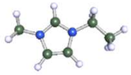
154.2899 Å^3</td>
<td>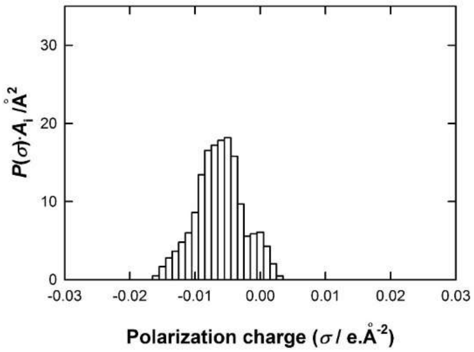
</td>
</tr>
<tr>
<td>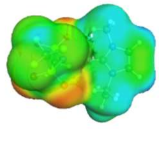
EmimTFSI
conformer 1
ΔE_d = 297.3 kJ·mol^{-1}</td>
<td>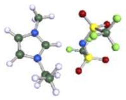
386.5976 Å^3</td>
<td>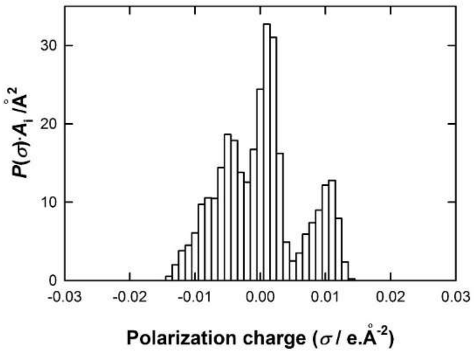
</td>
</tr>
</table>

<table>
<tr>
<td>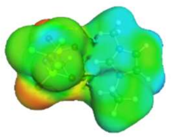
EmimTFSI
conformer 2</td>
<td>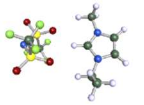
376.8248 Å^3</td>
<td>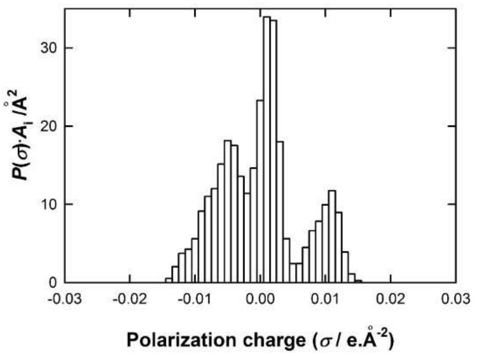
</td>
</tr>
<tr>
<td>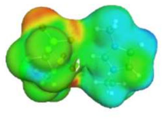
EmimTFSI
conformer 3</td>
<td>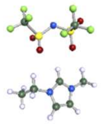
371.8710 Å^3</td>
<td>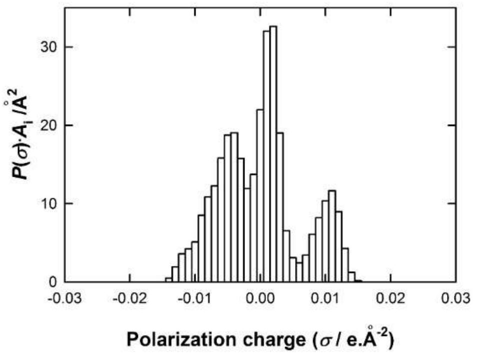
</td>
</tr>
<tr>
<td>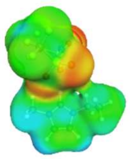
EmimTFSI
conformer 4</td>
<td>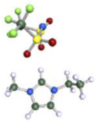
385.8893 Å^3</td>
<td>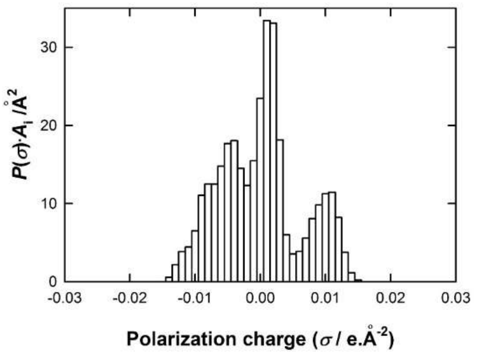
</td>
</tr>
<tr>
<td>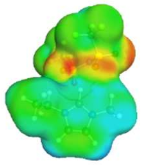
EmimTFSI
conformer 5</td>
<td>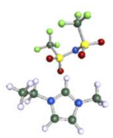
378.0977 Å^3</td>
<td>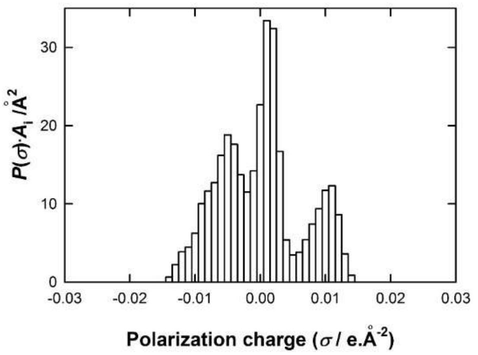
</td>
</tr>
</table>

<table>
<tr>
<td>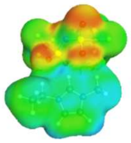
EmimTFSI
conformer 6</td>
<td>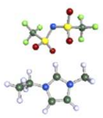
383.2116 Å^3</td>
<td>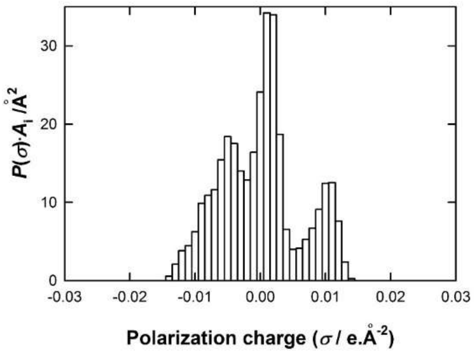
</td>
</tr>
<tr>
<td>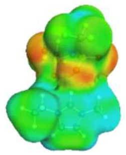
EmimTFSI
conformer 7</td>
<td>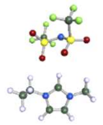
381.1088 Å^3</td>
<td>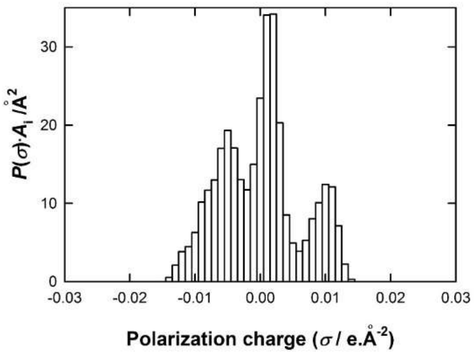
</td>
</tr>
<tr>
<td>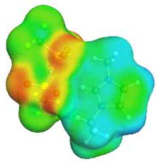
EmimTFSI
conformer 8</td>
<td>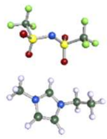
383.9792 Å^3</td>
<td>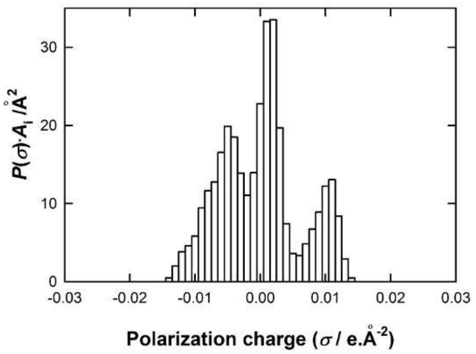
</td>
</tr>
<tr>
<td>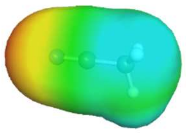
ACN</td>
<td>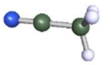
64.0469 Å^3</td>
<td>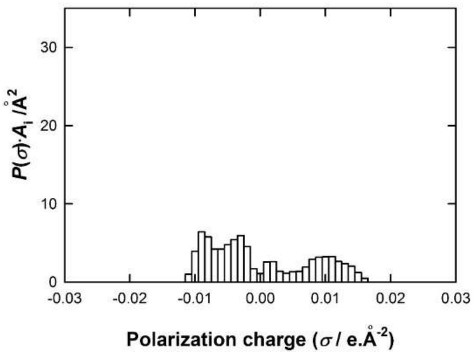
</td>
</tr>
</table>

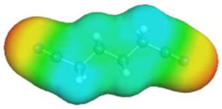<br>ADN<br>Conformer 1	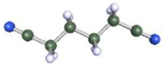<br>155.6710 Å^3	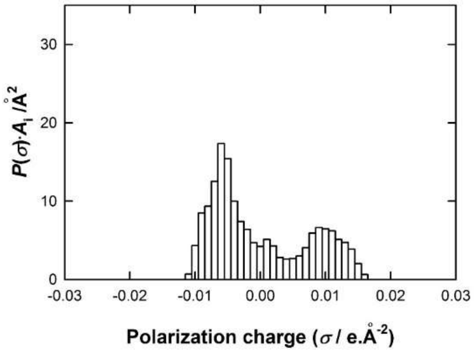
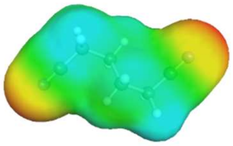<br>ADN<br>Conformer 2	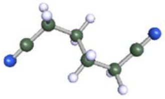<br>155.6680 Å^3	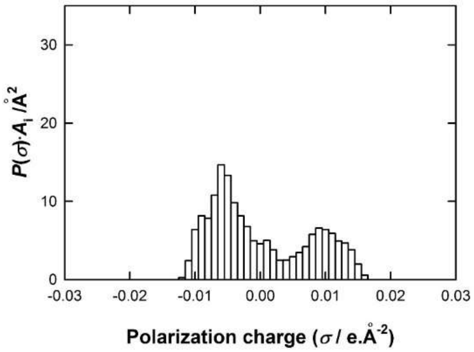
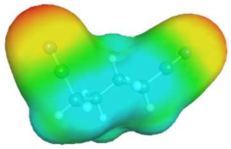<br>ADN<br>Conformer 3	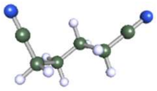<br>155.5776 Å^3	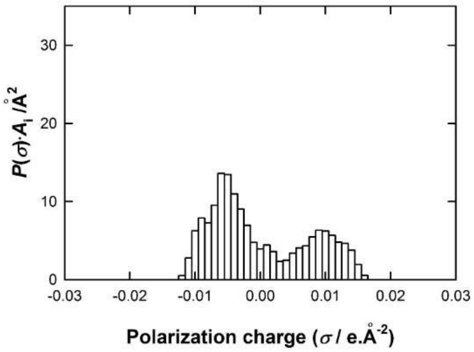
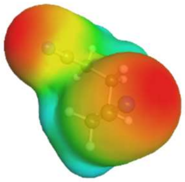<br>ADN<br>Conformer 4	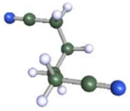<br>156.2771 Å^3	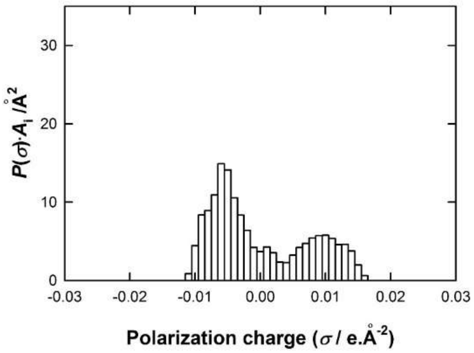

<table>
<tr><td>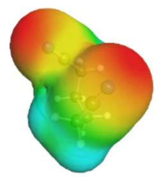
ADN
Conformer 5</td><td>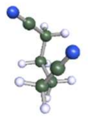
156.1704 Å^3</td><td>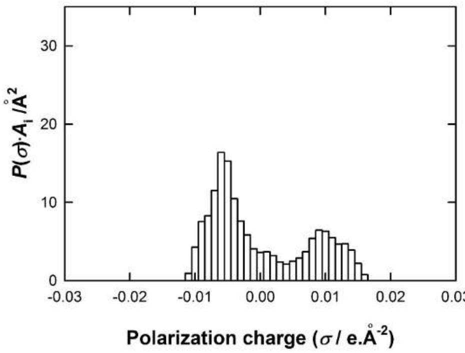
</td></tr>
<tr><td>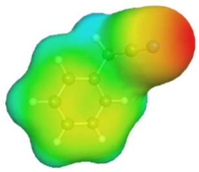
Ph-CN
Conformer 1</td><td>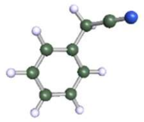
159.2185 Å^3</td><td>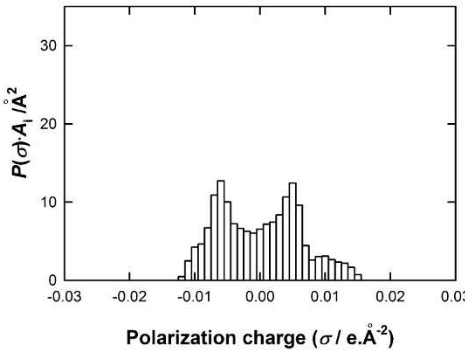
</td></tr>
<tr><td>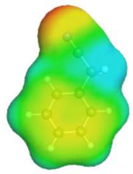
Ph-CN
Conformer 2</td><td>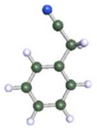
157.8790 Å^3</td><td>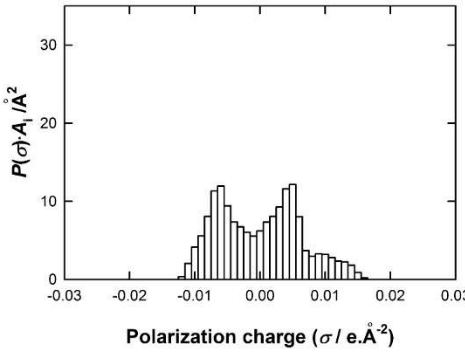
</td></tr>
</table>

We also focused our attention on the charge distribution of carbon and nitrogen atoms in the carbon nitrogen triple bonds, which seems to be nearly identical for each investigated nitrile-based solvent. Please note that in the case of ADN, the partial charge distribution was identical into each atom of each cyano group. This was done thanks to ab-initio and density functional theory (DFT, COSMO-RS solvation model) calculations using Turbomole 7.0 programme package.

Atomic populations from total density of cyano moieties:

Atom	Charge
ACN	
N	-0.31763
C	0.27123
ADN	
N	-0.31286
C	0.27503
Ph-ACN	
N	-0.31554
C	0.28409

Table S2. Experimental conductivity data of Ph-ACN and ADN based electrolytes as a function of the Et4NBF4 salt concentration up to its solubility limit at 25 °C as measured in a glovebox environment.

ADN		Ph-ACN	
[Et_4NBF_4] / M	σ / mS·cm^{-1}	[Et_4NBF_4] / M	σ / mS·cm^{-1}
0	0	0	0
0.1	1.16	0.1	0.93
0.2	1.98	0.2	1.73
0.3	2.63	0.3	2.42
0.4	3.15	0.4	2.99
0.5	3.61	0.5	3.44
0.6	4.05	0.6	3.74
0.7	4.30	0.65	3.84

Standard uncertainties u are $u(T)$ = 0.5 °C, u([Et_4NBF_4]) = 0.02·[Et_4NBF_4] and $u(\sigma) = 0.03 \cdot \sigma$.

Table S3. Experimental physical properties as a function of the temperature of pure solvents and selected Et4NBF4-based electrolytes at 101 kPa.

T / °C	ACN		1 M Et_4NBF_4 in ACN		
	ρ / g·cm^{-3}	η / mPa·s	ρ / g·cm^{-3}	η / mPa·s	σ / mS·cm^{-1}
-20	-	-	-	-	29.80
-15	-	-	-	-	31.86
-10	-	-	-	-	33.93
-5	-	-	-	-	36.03
0	-	-	-	-	38.13
5	0.7990	0.430	0.8670	0.785	40.24
10	0.7935	0.410	0.8622	0.746	42.36
15	0.7880	0.391	0.8574	0.707	44.47
20	0.7824	0.374	0.8525	0.668	46.59
25	0.7769	0.359	0.8476	0.639	48.70
30	0.7714	0.345	0.8427	0.611	50.81
35	0.7659	0.332	0.8377	0.591	52.91
40	0.7604	0.320	0.8328	0.575	55.01
45	0.7548	0.309	0.8278	0.550	57.09
50	0.7493	0.299	0.8228	0.524	59.16
55	0.7438	0.289	0.8179	0.501	61.22
60	0.7383	0.281	0.8128	0.481	63.27

Standard uncertainties *u* are $u(T)$ = 0.1 °C, u([Et_4NBF_4]) = 0.01·[Et_4NBF_4], $u(\rho) = 0.005 \cdot \rho$, $u(\eta) = 0.03 \cdot \eta$, $u(\sigma) = 0.01 \cdot \sigma$ and $u(p)$ = 2 kPa.

Table S3. Continued …

T / °C	ADN		0.7 M Et_4NBF_4 in ADN		
	ρ / g·cm^{-3}	η / mPa·s	ρ / g·cm^{-3}	η / mPa·s	σ / mS·cm^{-1}
-40	-	-	-	-	0.0003
-35	-	-	-	-	0.0002
-30	-	-	-	-	0.0002
-25	-	-	-	-	0.0002
-20	-	-	-	-	0.0002
-15	-	-	-	-	0.0003
-10	-	-	-	-	0.0003
-5	-	-	-	-	0.0005
0	-	-	-	-	0.0023
5	0.9740	11.80	0.9963	15.85	2.16
10	0.9702	9.900	0.9927	13.09	2.54
15	0.9664	8.413	0.9890	10.95	2.97
20	0.9627	7.224	0.9853	9.304	3.45
25	0.9589	6.272	0.9817	7.996	3.98
30	0.9551	5.499	0.9780	6.936	4.57
35	0.9514	4.858	0.9744	6.080	5.20
40	0.9476	4.328	0.9707	5.368	5.89
45	0.9439	3.885	0.9671	4.784	6.64
50	0.9402	3.509	0.9635	4.291	7.44
55	0.9364	3.190	0.9599	3.874	8.31
60	0.9327	2.919	0.9563	3.519	9.23
65	0.9290	2.688	0.9527	3.217	10.21
70	0.9253	2.490	0.9491	2.958	11.26
75	0.9216	2.319	0.9455	2.734	12.36
80	0.9179	2.170	0.9420	2.541	13.53

Standard uncertainties u are $u(T)$ = 0.1 °C, u([Et_4NBF_4]) = 0.01·[Et_4NBF_4], $u(\rho) = 0.005\cdot \rho$, $u(\eta) = 0.03\cdot \eta$, $u(\sigma) = 0.01\cdot\sigma$ and $u(p)$ = 2 kPa.

Table S3. Continued …

T / °C	Ph-ACN		0.65 M Et_4NBF_4 in Ph-ACN		
	ρ / g·cm^{-3}	η / mPa·s	ρ / g·cm^{-3}	η / mPa·s	σ / mS·cm^{-1}
-40	-	-	-	-	0.52
-35	-	-	-	-	0.70
-30	-	-	-	-	0.89
-25	-	-	-	-	1.13
-20	-	-	-	-	1.22
-15	-	-	-	-	1.40
-10	-	-	-	-	1.60
-5	-	-	-	-	1.84
0	-	-	-	-	2.10
5	0.7990	2.807	1.0451	4.572	2.40
10	0.7935	2.536	1.0412	4.016	2.73
15	0.7880	2.300	1.0373	3.558	3.11
20	0.7824	2.093	1.0334	3.186	3.53
25	0.7769	1.910	1.0295	2.884	4.00
30	0.7714	1.761	1.0256	2.624	4.53
35	0.7659	1.584	1.0217	2.408	5.13
40	0.7604	1.489	1.0178	2.229	5.80
45	0.7548	1.365	1.0138	2.078	6.54
50	0.7493	1.263	1.0099	1.949	7.37
55	0.7438	1.172	1.0060	1.839	8.29
60	0.7383	1.089	1.0021	1.743	9.31
65	0.7327	1.015	0.9982	1.660	10.46
70	0.7272	0.947	0.9943	1.588	11.72
75	0.7217	0.886	0.9904	1.524	13.13
80	0.7162	0.830	0.9865	1.454	14.68

Standard uncertainties u are $u(T)$ = 0.1 °C, u([Et_4NBF_4]) = 0.01·[Et_4NBF_4], $u(\rho) = 0.005\cdot \rho$, $u(\eta) = 0.03\cdot \eta$, $u(\sigma) = 0.01\cdot\sigma$ and $u(p)$ = 2 kPa.

Table S4. Experimental conductivity data (σ / mS·cm^{-1}) of investigated Ph-ACN + EmimTFSI blends as a function of the composition, expressed in IL mole fraction, xIL and IL concentration in mol.L-1, and the temperature at 101 kPa.

T / °C	C_{IL} /M	0.8	1.4	1.9	2.3	2.7	3.0	3.2	3.5	3.7	4.0
	x_{IL}	0.1000	0.2000	0.3070	0.3800	0.4990	0.6000	0.7000	0.7920	0.8700	1.0000
-20		2.51	2.87	2.52	2.60	2.28	1.65	1.73	1.50	1.27	1.08
-15		3.02	3.53	3.22	3.33	2.97	2.24	2.32	2.05	1.75	1.50
-10		3.57	4.26	4.01	4.15	3.78	2.94	3.01	2.72	2.34	2.03
-5		4.16	5.06	4.89	5.09	4.70	3.77	3.81	3.51	3.05	2.66
0		4.79	5.92	5.87	6.13	5.74	4.72	4.72	4.43	3.87	3.41
5		5.45	6.84	6.94	7.28	6.89	5.80	5.75	5.47	4.81	4.27
10		6.14	7.82	8.10	8.52	8.15	7.00	6.88	6.64	5.88	5.25
15		6.86	8.86	9.35	9.87	9.52	8.33	8.12	7.94	7.07	6.35
20		7.60	9.93	10.67	11.30	10.99	9.77	9.47	9.37	8.39	7.57
25		8.36	11.06	12.06	12.83	12.57	11.34	10.91	10.91	9.83	8.90
30		9.14	12.22	13.52	14.44	14.23	13.01	12.45	12.57	11.38	10.36
35		9.94	13.41	15.05	16.13	15.98	14.79	14.07	14.34	13.05	11.92
40		10.75	14.64	16.64	17.89	17.82	16.67	15.78	16.22	14.83	13.59
45		11.56	15.90	18.27	19.72	19.73	18.65	17.57	18.20	16.71	15.37
50		12.39	17.18	19.96	21.61	21.71	20.71	19.43	20.28	18.70	17.24
55		13.23	18.48	21.69	23.57	23.75	22.86	21.35	22.44	20.78	19.21
60		14.06	19.79	23.46	25.57	25.86	25.09	23.34	24.69	22.95	21.27
65		14.91	21.13	25.26	27.62	28.02	27.38	25.38	27.01	25.20	23.42
70		15.75	22.47	27.10	29.72	30.22	29.74	27.47	29.41	27.53	25.64
75		16.60	23.83	28.96	31.86	32.48	32.17	29.61	31.87	29.94	27.94
80		17.44	25.19	30.84	34.03	34.77	34.64	31.79	34.40	32.41	30.30

Standard uncertainties u are $u(T)$ = 0.1 °C, $u(x_{IL}) = 2\cdot10^{-4}$, $u(\sigma) = 0.01\cdot\sigma$ and $u(p)$ = 2 kPa.

Table S5. Experimental physical properties as a function of the temperature of pure IL and 2.7 M EmimTFSI in Ph-ACN electrolyte at 101 kPa.

T / °C	EmimTFSI		2.7 M EmimTFSI in Ph-ACN	
	ρ / g·cm^{-3}	η / mPa·s	ρ / g·cm^{-3}	η / mPa·s
5	1.5389	76.48	1.3753	20.20
10	1.5338	61.06	1.3705	16.76
15	1.5287	49.31	1.3657	14.12
20	1.5236	40.56	1.3609	12.04
25	1.5185	33.85	1.3561	10.40
30	1.5135	28.81	1.3514	9.069
35	1.5084	24.51	1.3466	7.979
40	1.5034	21.40	1.3418	7.083
45	1.4984	18.68	1.3371	6.334
50	1.4934	16.48	1.3324	5.697
55	1.4885	14.66	1.3277	5.158
60	1.4835	13.04	1.3230	4.693
65	1.4786	11.71	1.3183	4.298
70	1.4737	10.65	1.3136	3.957
75	1.4688	9.624	1.3090	3.658
80	1.4639	8.901	1.3043	3.396

Standard uncertainties u are $u(T)$ = 0.1 °C, u([IL]) = 0.01·[IL], $u(\rho) = 0.005\cdot\rho$, $u(\eta) = 0.03\cdot\eta$ and $u(p)$ = 2 kPa.

Table S6. Optimized structure of investigated Emim+ cation + Ph-ACN clusters.

4 Ph-ACN + 1 Emim$^+$	5 Ph-ACN + 1 Emim$^+$
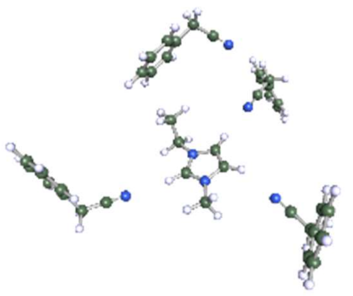	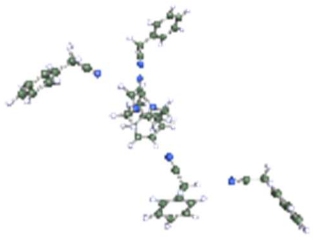

Table S7. Electrochemical stability windows of selected electrolytes.

Electrolyte	Ec *vs.* Ag/Ag$^+$ (V)	Ea *vs.* Ag/Ag$^+$ (V)	ESW (V)
**0.65 M Et4NBF4 in Ph-ACN**	-2.39	+1.90	4.29
**2.7 M Emim-TFSI in Ph-ACN**	-2.27	+2.67	4.94
**Pure Emim-TFSI**	-1.89	+2.51	4.40

Table S8. Structure, HOMO and LUMO energies of each selected solvent, ion and salt.

Species	Structure	HOMO (eV)	LUMO (eV)
BF_4^-	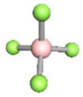	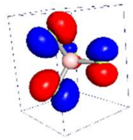<br>-4.231	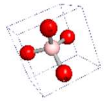<br>+0.2426
Et_4N^+	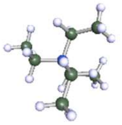	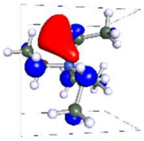<br>-13.461	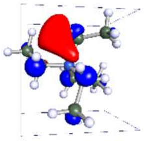<br>-3.200
Et_4NBF_4	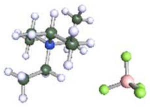	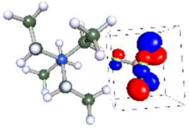<br>-7.255	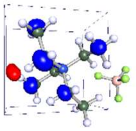<br>-0.746
$TFSI^-$	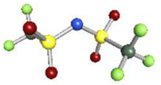	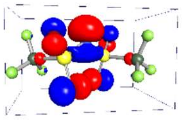<br>-4.188	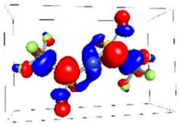<br>+3.347
$Emim^+$	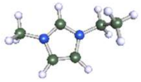	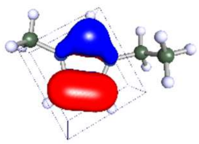<br>-11.807	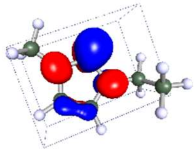<br>-5.165
EmimTFSI	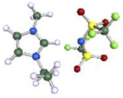	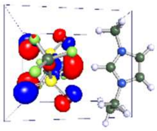<br>-7.377	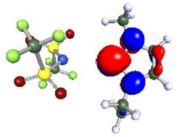<br>-1.951

ACN	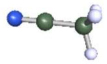	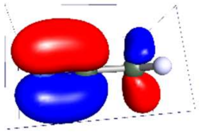<br>-9.080	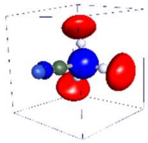<br>+0.533
ADN	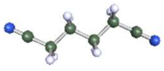	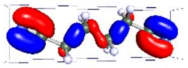<br>-9.089	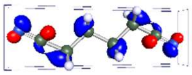<br>-0.171
Ph-ACN	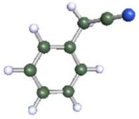	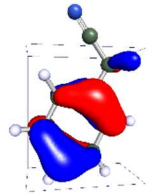<br>-7.178	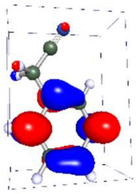<br>-0.871

Table S9. Electrolyte resistance (Rs), electrolyte series resistance (ESR), and specific capacitances (C), before and after floating tests for both the 0.65 M Et_4NBF_4 in Ph-ACN (a), and 2.7 M EmimTFSI in Ph-ACN (b)

a)

	Before floating			After floating		
**0.65 M Et_4NBF_4 in Ph-ACN**	**R_s (Ohm)**	**ESR (Ohm)**	**C (F/g)**	**R_s (Ohm)**	**ESR (Ohm)**	**C (F/g)**
2.7 V	4.77	~8	17.86	5.8	~13	20.45
3.0 V	6.61	~10	17.69	7.76	~22	20.84
3.2 V	4.7	~11	17.46	8.5	~32	18.09

b)

	Before floating			After floating		
**EmimTFSI in Ph-ACN**	**R_s (Ohm)**	**ESR (Ohm)**	**C (F/g)**	**R_s (Ohm)**	**ESR (Ohm)**	**C (F/g)**
2.7 V	3	~6	20.22	3.01	~7	23.48
3.0 V	3	~6	22.34	3.5	~10	23.72
3.2 V	4.2	~6	23.39	5.1	~15	22.84
